# A Neural Mechanism in the Human Orbitofrontal Cortex for Preferring High-Fat Foods Based on Oral Texture

**DOI:** 10.1523/JNEUROSCI.1473-23.2023

**Published:** 2023-11-22

**Authors:** Putu A. Khorisantono, Fei-Yang Huang (黃飛揚), Michael P. F. Sutcliffe, Paul C. Fletcher, I. Sadaf Farooqi, Fabian Grabenhorst

**Affiliations:** ^1^Department of Physiology, Development and Neuroscience, University of Cambridge, Cambridge CB2 3DY, United Kingdom; ^2^Department of Experimental Psychology, University of Oxford, Oxford OX1 3TA, United Kingdom; ^3^Department of Engineering, University of Cambridge, Cambridge CB2 1PZ, United Kingdom; ^4^Wellcome Trust-MRC Institute of Metabolic Science, Addenbrooke's Hospital, Cambridge CB2 0QQ, United Kingdom

**Keywords:** dietary fat, oral food processing, neuroeconomics, preference, reward value

## Abstract

Although overconsumption of high-fat foods is a major driver of weight gain, the neural mechanisms that link the oral sensory properties of dietary fat to reward valuation and eating behavior remain unclear. Here we combine novel food-engineering approaches with functional neuroimaging to show that the human orbitofrontal cortex (OFC) translates oral sensations evoked by high-fat foods into subjective economic valuations that guide eating behavior. Male and female volunteers sampled and evaluated nutrient-controlled liquid foods that varied in fat and sugar (“milkshakes”). During oral food processing, OFC activity encoded a specific oral-sensory parameter that mediated the influence of the foods' fat content on reward value: the coefficient of sliding friction. Specifically, OFC responses to foods in the mouth reflected the smooth, oily texture (i.e., mouthfeel) produced by fatty liquids on oral surfaces. Distinct activity patterns in OFC encoded the economic values associated with particular foods, which reflected the subjective integration of sliding friction with other food properties (sugar, fat, viscosity). Critically, neural sensitivity of OFC to oral texture predicted individuals' fat preferences in a naturalistic eating test: individuals whose OFC was more sensitive to fat-related oral texture consumed more fat during *ad libitum* eating. Our findings suggest that reward systems of the human brain sense dietary fat from oral sliding friction, a mechanical food parameter that likely governs our daily eating experiences by mediating interactions between foods and oral surfaces. These findings identify a specific role for the human OFC in evaluating oral food textures to mediate preference for high-fat foods.

**SIGNIFICANCE STATEMENT** Fat and sugar enhance the reward value of food by imparting a sweet taste and rich mouthfeel but also contribute to overeating and obesity. Here we used a novel food-engineering approach to realistically quantify the physical-mechanical properties of high-fat liquid foods on oral surfaces and used functional neuroimaging while volunteers sampled these foods and placed monetary bids to consume them. We found that a specific area of the brain's reward system, the orbitofrontal cortex, detects the smooth texture of fatty foods in the mouth and links these sensory inputs to economic valuations that guide eating behavior. These findings can inform the design of low-calorie fat-replacement foods that mimic the impact of dietary fat on oral surfaces and neural reward systems.

## Introduction

Oral food processing involves a chain of sensory and neural events in which a food's physical structure elicits oral sensations and subjective valuations that guide eating behavior. For example, foods high in sugar and fat typically produce a characteristic “mouthfeel” in the form of a sweet taste and thick, smooth texture. These sensory signals are thought to contribute to the reward value of food, to near-universal preferences for foods high in sugar and fat, and to the development of obesity (Alonso-Alonso et al., 2015; Carreiro et al., 2016; Rolls, 2020; Huang et al., 2021).

Specific brain areas, such as the orbitofrontal cortex (OFC), play key roles in assigning reward values to foods based on sensory inputs (Grabenhorst and Rolls, 2011; Rangel, 2013; Rolls, 2016; Morys et al., 2022). Studies in decision neuroscience identified neural responses to visual food stimuli that reflected valuation and related decision-making based on food attributes, such as nutrients, healthiness, and tastiness, specifically in the OFC and ventromedial PFC (Hare et al., 2009, 2011; Grabenhorst et al., 2013; Tang et al., 2014; Suzuki et al., 2017). Investigations in sensory neuroscience identified single-neuron activities in macaques and neuroimaging signals in humans that reflect oral food properties, including taste, odor, flavor, oral temperature, and texture, specifically in the OFC, insula, amygdala, and somatosensory cortex (de Araujo and Rolls, 2004; Small et al., 2004, 2008; Guest et al., 2007; Grabenhorst et al., 2008, 2010; Eldeghaidy et al., 2011; Grabenhorst and Rolls, 2014; Avery et al., 2020; Rolls, 2020). Despite these advances, the neural mechanisms that link the physical, material properties of foods to oral sensations, reward valuations, and economic preferences remain poorly understood.

Although sugar is sensed directly by taste receptors (Yarmolinsky et al., 2009), the mechanism for oral-fat sensing remains a topic of debate (Mattes, 2009). Substantial evidence points to a somatosensory, oral-texture mechanism for fat sensing (Kokini, 1987; Rolls, 2016). Fat-like textures elicit a smooth, creamy mouthfeel (Chojnicka-Paszun et al., 2012; Upadhyay et al., 2020) and activate neural sensory and reward systems (de Araujo and Rolls, 2004; Grabenhorst et al., 2010; Grabenhorst and Rolls, 2014; Rolls et al., 2018). Traditionally, viscosity has been considered a key oral-texture parameter important in fat sensing (Kokini, 1987; Mela, 1988; Stokes et al., 2013). Viscosity is a material, “bulk” food property that is measured as resistance to flow using rheological techniques (Bourne, 2002; Upadhyay et al., 2020). However, recent advances in food science identified the coefficient of sliding friction (CSF) as a distinct texture parameter important in oral lubrication. Unlike viscosity, sliding friction (i.e., the contact force measured between oral surfaces sliding against each other) results from dynamic interaction of food with oral surfaces, which is measured using tribological techniques that are sensitive to frictional rather than flow properties (Chen and Stokes, 2012; Stokes et al., 2013; Laguna et al., 2017). Separate neurons in primate OFC and insula encode CSF and viscosity (Rolls et al., 1999, 2003, 2018; Verhagen et al., 2003). An important question, therefore, is to what extent these two associated but distinct parameters — viscosity and sliding friction — contribute to oral-fat sensing and its rewarding properties.

Here we combined functional neuroimaging and behavioral experiments in human volunteers with food-engineering methods that quantified food textures in a realistic manner on biological oral surfaces (Huang et al., 2021). We investigated the neural mechanisms that link the oral processing of foods high in fat and sugar to subjective sensations and preferences. We hypothesized that CSF and viscosity would partly mediate the influence of fat content on subjective food valuations. We also hypothesized that neural reward systems process both the physical, oral-texture parameters of foods and the subjective sensations and valuations elicited by them. Finally, we sought to determine the predictive value of these measures by examining whether neural sensitivity to oral texture predicted subjects' eating behavior in a naturalistic context.

## Materials and Methods

### Participants

A total of 22 healthy volunteers participated in the full study (19-36 years old, mean age 25 years, SD = 4.8 years; 15 males, based on written self-report). Of 23 participants scanned, one dropped out during the first scanning session and was therefore excluded from the study. We initially aimed for a sample size of 24 participants, based on expected effect sizes from prior studies. However, it was not possible to complete data collection for all planned subjects because of Covid-related lockdown and restrictions in the United Kingdom at the time the study was conducted. Notably, each participant was scanned on 2 d to maximize the amount of data for decoding analyses. The sample size is comparable with previous papers in the field (Suzuki et al., 2017; DiFeliceantonio et al., 2018; Avery et al., 2020). Participants had a mean weight of 72.8 ± 14.2 kg and a mean BMI of 23.4 ± 2.56 kg/m^2^ (4 participants had a BMI between 25 and 29 kg/m^2^). Participants confirmed that they were not currently dieting, nonobese, and had no history of neurologic or psychiatric conditions. In post-study questionnaires, participants reported on average a liking for sweet and fatty foods (6.44 ± 1.92 and 6.28 ± 2.28, respectively; liking scales ranging from 0 to 10, labeled “not at all” and “very much,” respectively) and reported their milk preference for different fat levels (stated preferences: whole milk: 38.1% of participants; semi-skimmed milk: 33.3%; skimmed milk: 14.3%; nondairy milk: 14.3%). All participants provided written consent based on an information sheet. The study was approved by the Cambridge Local Research Ethics Committee. The different parts of the study were conducted at the Department of Physiology, Development and Neuroscience, University of Cambridge, at the Wolfson Brain Imaging Center, and at the Wellcome-MRC Institute of Metabolic Science Translational Research Facility.

### Study design

**Figure 1.**
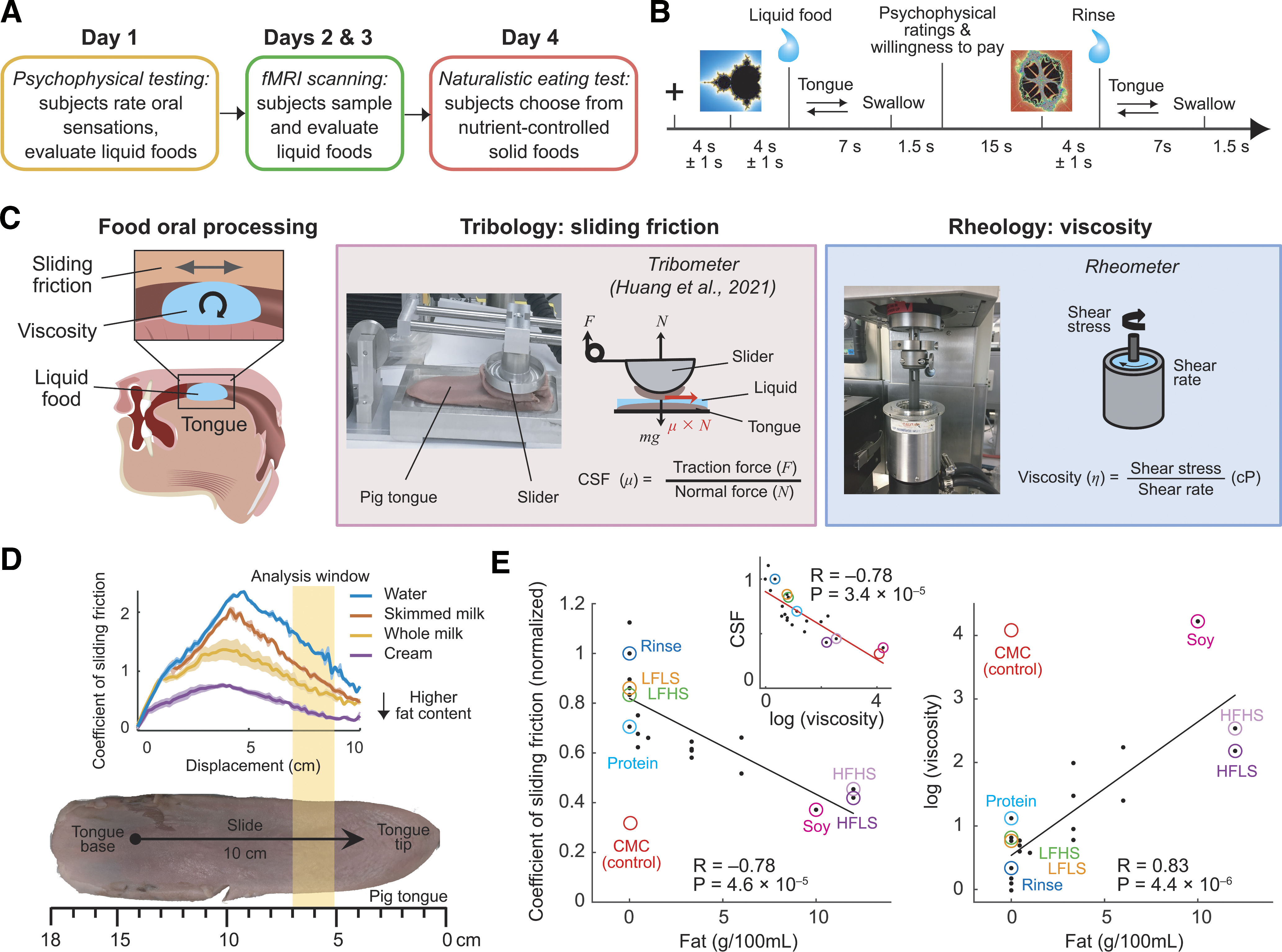
Study design and oral-sensory measurements for experimental foods. ***A***, Sequence of experiments in each participant. ***B***, Trial structure for controlled sampling and oral processing of liquid foods in psychophysical and fMRI experiments. Liquid foods were delivered orally under computer control via peristaltic pumps. Subjects performed a pretrained left-right (or right-left, alternating randomly trial by trial) tongue movement guided by a visual cue to standardize oral processing. ***C***, Sliding friction and viscosity as key texture variables in food oral processing. Left, Sliding friction results from oral-surface movements, with liquid foods acting as lubricant; viscosity is a “bulk” material food property. Middle, Custom tribometer measures the CSF of experimental liquid foods in a biologically realistic manner using sliding pig tongues. Right, Rotational rheometer measures the viscosity of liquid foods. ***D***, Validation of tribological measures of sliding friction in a series of basic fat-containing liquids. CSF reflects liquids' fat content. ***E***, Linear regression of CSF and viscosity on fat content in a large set of experimental liquid foods. Colored foods were used in the present experiments. Inset, Relationship between CSF and viscosity. HPHS, Low-fat, high-sugar, high-protein; Soy, soy-based HFHS; CMC, low-fat, high-sugar thickened with carboxymethyl cellulose. CMC represents a nonfat artificially thickened control stimulus that was excluded from the regression.

To investigate behavioral and neural mechanisms underlying food preferences during oral food processing, we tested each participant (*N* = 22) in a series of experiments (see Fig. 1*A*). First, in a psychophysical test (day 1), subjects repeatedly sampled liquid food stimuli with well-controlled sensory and nutrient components and provided psychophysical ratings of food sensations; they also placed monetary bids to consume the foods in an economic auction-like task (Becker et al., 1964). Second, subjects participated in two fMRI scanning sessions (days 2 and 3) in which these tasks were repeated in the MRI scanner, including the oral sampling of the liquid foods (two scanning days were required to obtain sufficient data for multivoxel pattern fMRI analysis). Third, subjects participated in an *ad libitum* eating test (day 4) in which they made consumption choices for nutrient-controlled solid foods under naturalistic, life-like conditions. This design allowed us to investigate behavioral and neural responses to liquid foods under controlled experimental conditions and during realistic eating behavior involving solid foods.

Importantly, to orally process the liquid foods, subjects were trained to perform a standardized, left-right (or, in random alternation, right-left) tongue movement guided by a dynamic visual cue moving at a defined speed (replaced by a static left/right-pointing arrow for MRI scanning), and then hold their tongue still for several seconds before swallowing the liquids. This manipulation ensured (1) distribution of liquids around the oral cavity (Grabenhorst et al., 2010), (2) standardized oral mechanical and sensory stimulation across stimuli and subjects, and (3) oral sliding-friction stimulation at a defined speed that matched our tribology measurements (see below). This manipulation was important given our focus on oral texture and the importance of tongue movements in food-texture sensing (de Wijk et al., 2003, 2011; Koc et al., 2013).

### Design of food stimuli

We designed a set of dairy-based liquid food stimuli (“milkshakes”) with controlled nutrient and energy content to produce variation in sensory food stimulation, subjective sensations and economic valuations (see Table 1). A 2 × 2 factorial design with sugar and fat as factors defined the following basic stimuli: (1) a low-fat, low-sugar liquid (LFLS), (2) a high-fat, low-sugar liquid (HFLS), (3) a low-fat, high-sugar liquid (LFHS), and (4) a high-fat, high-sugar liquid (HFHS). Energy content was lowest for the LFLS stimulus, intermediate for the HFLS and LFHS stimuli, and highest in the HFHS stimulus. In addition, we included (5) a low-fat, high-sugar and high-protein stimulus to assess fat-like texture produced by protein, (6) a HFHS based on soy rather than dairy milk to include both animal- and plant-based fat stimuli, and (7) a LFHS that was thickened by adding carboxymethyl cellulose (a food-thickening agent used in the food industry) to produce a fat-like texture. Following previous studies (de Araujo et al., 2003; Grabenhorst et al., 2008, 2010), “artificial saliva” was used as a rinse. This rinse stimulus contained the main ionic components of saliva (25 mm KCl + 2.5 mm NaHCO_3_). All stimuli except artificial saliva were vanilla-flavored (Sainsbury's Madagascan vanilla extract); temperature during the experiments was held constant by the use of ice-packs. In the first testing session, subjects were trained to associate a distinct, abstract visual cue with each of the eight liquid food stimuli by performing the psychophysical rating task in which visual cues were presented before liquid delivery on each trial. Subjects did not receive any feedback or explicit information regarding the nutrient content of the liquid food stimuli. The visual conditioned stimuli were presented before the liquid food stimuli were delivered in the psychophysical task and during MRI scanning to reduce participants' uncertainty about which liquid would be delivered a given trial. Visual cues were well separated from the liquid delivery by a jittered interstimulus interval of 4 ± 1 s. We used the same set of eight visual cues across subjects but changed the association between specific visual cues and liquids to avoid systematic visual confounds. We did not include an identification task to examine whether subjects could reliably recall the visual cues associated with the stimuli, although stimulus ratings were consistent between pretesting and scanning sessions and across the two scanning sessions (correlation across stimuli between pretest and scanning sessions: sweetness: *R* = 0.937, *p* = 0.0018; thickness: *R* = 0.978, *p* = 0.0001; correlation across stimuli between two scanning sessions: sweetness: *R* = 0.997, *p* = 9.1 × 10^−7^; thickness: *R* = 0.977, *p* = 0.0001).

**Table 1.** Composition of experimental liquid food stimuli*^a^*

	LFLS	HFLS	LFHS	HFHS	HPHS	Soy	CMC	Rinse
Fat	0	12	0	12	0	10	0	0
Sugar	3.3	3.3	6.7	6.7	6.7	6.7	6.7	0
Protein	2.8	2.8	1.8	2.8	6.8	2.5	1.8	0
Calories	0.22	1.33	0.35	1.46	1.13	1.29	0.35	0
Viscosity	2.15	8.86	2.26	12.64	3.07	68.2	59.26	1.4
CSF	0.86	0.45	0.83	0.45	0.70	0.37	0.31	1

*^a^*Fat, sugar, protein: g/100 ml; calories: kcal/100 ml; viscosity: centipoise, CSF: normalized to water.

### Delivery of liquid food stimuli

In order to deliver the seven liquid food stimuli and the rinse stimulus while the participant underwent MRI scanning, custom-made computer-controlled peristaltic pumps were used. The pumps were set up in the scanner control room, where the experimenter, the radiographer, and the testing laptop were located. Food-grade silicone tubing was connected to each pump output and threaded through the connecting aperture into the scanner room. Participants held a soft, custom-made mouthpiece (a modified infant food feeder; Losuya) between their lips that connected the eight tubes without mixing the stimuli. The pumps were the same as used in a previous human fMRI experiment involving similar liquid food stimuli (Zangemeister et al., 2016). The pumps were controlled by the laptop with the use of a National Instruments card. The rotational speed of the pump rotor was constant within each pump. We controlled the volume of the liquid delivered by specifying the length of time the pump was activated. From previous studies involving liquid reward delivery in the fMRI (Grabenhorst et al., 2010; Zangemeister et al., 2016), we aimed to deliver between 0.75 and 1 ml to participants as this was shown to be sufficient to activate neural systems processing taste, flavor, and texture. We carefully and regularly calibrated the pumps to ensure constant and matched delivery volumes across stimuli.

### Instructed tongue movements

In order to ensure even dispersal of the stimuli and rinse, and to ensure that texture-related subjective ratings were not confounded by variability in tongue movement, participants were instructed in how to move their tongue during the tasting period. In the behavioral pretesting task, a cursor appeared in the center of the screen during the tasting and rinse period. This cursor would move either to the left, then to the right before coming back to the center or to the right, then the left before coming back to the center, and participants were asked to move their tongue across the palate mirroring the cursor's movement. The direction in which the cursor moved was randomized for every trial and taste period such that the participant could not predict the direction before the appearance of the cursor. This task trained the participants to move their tongues with a specific speed and reduced the likelihood of results arising from differences in tongue movement. During the fMRI scanning task, the tongue movement cursor was replaced with a static arrow. This replacement reduced the need for participants to actively track the cursor movement across the screen with their eyes to minimize visual effects.

### Task structure for psychophysical testing and MRI scanning

We used a similar trial structure for psychophysical testing and MRI scanning. The event timing for the fMRI task is shown in Figure 1*B*. Each trial started with the display of one of the eight visual conditioned stimuli for 1.5 s, followed by an interstimulus interval during which a central fixation cross was displayed for 4 ± 1 s. The visual cue served to prepare the subjects for the subsequent delivery of a liquid food stimulus. Delivery of one of the food stimuli (chosen in a random permuted sequence) was cued by a colored fixation cross which stayed on the screen for 7 s. During this period, a cursor or arrowhead pointing to the left or right was presented next to the fixation spot for 1 s to guide the subject's tongue movement. As described above, subjects were pretrained to make one left-right tongue movement under visual guidance and then hold still until they were cued to swallow. After this 7 s tasting period, the letter S was presented for 1.5 s to cue subjects to swallow. After this period, two response periods followed in each of which a labeled visual analog rating scale was presented and subjects moved a bar to the appropriate point on the scale using a button box.

During psychophysical testing (day 1), subjects made five different rating responses by rating sweetness, thickness, oiliness, protein content, willingness to pay (WTP), and made a binary choice response whether or not the stimulus contained fat. For the rating scales, we used descriptors previously used in relation to taste and textural attributes, including sweetness, thickness, and oiliness (defined as an oil-like feel) (Szczesniak, 1963; Bourne, 2002; de Araujo and Rolls, 2004; Kadohisa et al., 2005). Subjects received written instructions with real-life examples in how to use these ratings scales to evaluate the food stimuli. Given our focus on oral-texture properties, the present manuscript focuses on analyzing ratings of sweetness, thickness, oiliness, and WTP. During MRI scanning, restrictions on scan time required a simplified design involving two types of trials in which different ratings were used in random alternation: subjects either rated the food stimuli on separate scales for sweetness and thickness (trial Type 1) or placed a WTP bid and made a binary choice about whether the food stimulus contained fat (trial Type 2). Each rating period was 4 s long. After the second rating period ended, a fixation cross was displayed for 4 ± 1 s, followed by the visual conditioned cue associated with the rinse stimulus, which was displayed for 1.5 s. The delivery and timing of the rinse period, including the tongue movement and swallowing periods, were identical to the liquid food delivery, except that no rating scales were used for the rinse stimulus. Thus, the trial ended with the swallowing of the rinse and was followed by an intertrial interval of 4 ± 1 s during which a fixation cross was displayed. A typical trial lasted ∼44 s (depending on the jittered periods).

MRI scanning was performed in 6 runs of ∼15 min each, to provide sufficient numbers of runs for training and testing for the multivariate fMRI data analysis. One subject was instead tested with 5 runs of ∼18 min. The 6 runs were performed on 2 separate days <1 week apart. Importantly, to create a balanced design for the multivariate analysis, each food stimulus was delivered 3 times within each run. Thus, in total each food stimulus was delivered 18 times. Occasional trials in which subjects reported that no stimulus was delivered (e.g., because of liquid backflow) were excluded from analysis. This general protocol and design have been used successfully in previous studies to investigate activations and their relation to subjective ratings in cortical areas (Grabenhorst et al., 2008, 2010; Grabenhorst and Rolls, 2014; Zangemeister et al., 2016).

### WTP measure

We measured the subjective, economic value of the food stimuli in a Becker-DeGroot-Marschak auction-like task (Becker et al., 1964), that has previously been used in functional neuroimaging and behavioral experiments with visual and liquid food stimuli (Plassmann et al., 2007; Zangemeister et al., 2016; Suzuki et al., 2017). Participants provided a bid of 0-10 credits on the liquid food stimulus they received on a given trial. At the beginning of the behavioral pretest, as well as at the beginning of each MRI scanning run, the participant received 100 credits endowment to spend on their bids. Using this endowment, participants placed bids on the liquid food stimulus they had just sampled on the same trial. Participants were instructed that they would bid to consume 250 ml of the just-sampled food after the experiment, and that their bid would be successful if it exceeded a computer-generated bid on the same trial (which was generated from a uniform distribution with replacement). We provided written instructions to the participants which explained that the best strategy was to bid exactly the amount that reflected their subjective value of the food stimulus; in that way, they would ensure to obtain the preferred food for the price they were willing to pay and avoid obtaining nonpreferred foods. Participants placed bids by moving a cursor on a WTP scale. When participants lost their bid, they lost none of their budget, although when they won, the computer-generated random price was deducted from their budget.

#### Rheological measurements of viscosity

We measured the viscosity of the experimental liquid food stimuli at the Rheology Center of the Department of Chemical Engineering, University of Cambridge. Measurements were performed using a Rheometric Scientific ARES controlled strain rheometer (TA Instruments) with the following Couette geometry: cup diameter = 34.0 mm, bob diameter = 32.0 mm, bob length = 34.0 mm. Viscosity was measured by carrying out shear-rate sweeps from 100-0.1 s^−1^ or 1 s^−1^ (reverse sweep) after allowing samples to equilibrate at the experimental temperature for 300 s. We measured the samples at different temperatures including 18.5°C (the temperature at which stimuli were delivered into the subject's mouth), 25°C, and 37.5°C (body temperature). We used measurements taken at 18.5°C for our data analyses, based on the temperature of the testing liquids (17 ± 2°C) measured during the experiment, which were kept constant with ice packs. When the liquid sample reached the determined temperature, the cup rotated first clockwise and then counterclockwise. In the time-sweeping measurement, the cup was rotated in a clockwise direction and the shear rate was fixed at 50 s^−1^; rheological properties of liquid stimuli have been shown at these settings to be related to oral viscosity evaluations (Wood, 1968).

#### Tribological measurements of the CSF

We measured the CSF for our liquid rewards at the Department of Engineering, University of Cambridge. To reflect realistic lubrication conditions in the oral cavity, we devised a custom-designed tribometer using pig tongues as biological contacting surfaces (see Fig. 1*C*), as described previously (Huang et al., 2021). The design involved a flat aluminum platform that held a fixed base consisting of the (nominally flat) pig tongue. An upper moving tongue-tip tissue was attached with superglue onto a dome-shaped slider of radius 100 mm. Thus, the top and bottom tissues contacted with a nominal point contact, avoiding any issues with alignment during the sliding process. The anatomically upper surface of each tongue was used for each of the two contacting surfaces. We mounted the dome-shaped slider using low-friction bushings onto a track containing two rails, pivoted at one end. We used a counterweight to balance out the weight of the rail elements of the track. Therefore, the load through the contact between the tongues was determined by the weight of the dome-shaped slider and tongue tip specimen (2.58 ± 0.07 N); these did not vary as the slider moved along the track. The slider was attached via a pulley to an Instron 5544 Universal Testing machine (Instron). We confirmed in preliminary tests that pulley friction was negligible. During testing, we loaded the moving tissue against the fixed base pig tongue with the testing liquids interfaced as lubricating layers. The Instron machine then imposed a fixed velocity (*v* = 16 mm/s) to the slider, while measuring the traction force using a load cell attached to the Instron machine. Accordingly, this design measured the sliding friction between the test liquids and the oral tissues; it thereby approximated biological oral sensing conditions. As we maintained a constant velocity during the test, according to Newton's First Law of Motion, as follows:

F−μN=0

$$\mu =\frac{traction\;force\;(F)}{loading\;force\;(N)}=\frac{traction\;force\;measured\;by\;the\;tensile\;machine}{total\;weight\;of\;the\;slider\;and\;the\;fixed\;tongue\;tip}$$

where *F* is the applied force (traction) by the Instron machine, *N* is the loading force perpendicular to the contact surface (normal force), and μ (the CSF) is the ratio of the traction force and the perpendicular loading force.

On the day before testing, we obtained fresh pig tongues from a local butcher (Leech & Sons) which we gently rinsed with water to remove residual blood and tissue fluids. We then placed the superficial 1 cm thick, anterior 18 cm of the tongues onto a flat contact surface that fitted onto the testing platform. We preserved the processed pig-tongue slices in isotonic saline buffer (PBS, 1×, pH 7.4) in a freezer under 4°C overnight. On the testing day, we prepared the contact surfaces by gluing one 18 cm tongue on the base platform and another tongue tip (5 cm) on the surface of the dome-shaped slider. We weighed the dome-shaped slider with the attached tongue tip to give the loading force for later calculation of the CSF. Before each measurement, both tongue surfaces were rinsed with 10 ml of PBS 3 times to remove residual testing liquids and hydrate the tongue surfaces. Next, we loaded 30 ml of the testing liquids between the pig tongues and pulled the slider from the posterior tongue bases forward to the anterior tongue tip (see Fig. 1*C*). Measurements were performed with testing liquids at room temperature. For practical reasons, we were unable to measure CSF at different temperatures because the measurements using fresh pig tongues were time-consuming and each pig tongue could only be used for a limited number of measurements before the surface degraded. The procedures were approved by the Departmental Safety Office, Department of Engineering, University of Cambridge, based on Control of Substances Hazardous to Health and biohazard risk assessments.

For the final testing of the stimuli, we measured each liquid with triplicate repeats using two pairs of pig tongues in opposite measuring orders to control for possible carryover effects (low-fat to high-fat liquids and reverse). We first averaged the triplicate measurements for each liquid and divided them by the corresponding loading force to obtain the CSF along the tongue surface. We selected the anterior 5-7 cm of the tongues as our analysis window because the mechanosensory receptors are located mainly within the anterior two-thirds of tongue (Ogawa et al., 1989; Toda and Taoka, 2002); more anterior tongue tips were too thin for stable measurements. Because inevitable variations in tongue conditions influenced the CSF measurement, we normalized for each pair of testing tongues the measured CSF with the CSF of water obtained from the same pair of tongues. This normalization adjusted the offset in absolute measurement because of variations of the tongues and provided comparable results between different tongue pairs. Finally, we averaged the two normalized CSF obtained from the different tongues for our analyses.

#### Naturalistic food-preference test

This test was performed at the Translational Research Facility of the Wellcome-MRC Institute for Metabolic Sciences at Addenbrooke's Hospital, Cambridge. It was possible to test 20 of the 22 subjects that participated in psychophysical testing and MRI scanning sessions also in this naturalistic food-preference test; 2 subjects did not return for this separate testing session. The stimulus design and testing procedure followed a previous study (van der Klaauw et al., 2016). We conducted an *ad libitum* eating test in which participants were allowed to choose freely and repeatedly between three curries that were approximately matched in terms of visual appearance and spicing but had varying nutrient compositions. The curries were based on the chicken korma from a previous study (van der Klaauw et al., 2016), except that the protein component was from a popular meat replacement (Quorn) to not exclude vegetarian participants. The nutrient compositions of the curries were designed such that they had three distinct fat and sugar levels, following a similar ratio to that of the liquid milkshakes used in the behavioral pretesting and fMRI component of the study (see Table 6). The curries were finely chopped and mixed to match their visual appearance. The test was conducted as follows.

After their second MRI scanning session, participants were invited to a fourth visit. Participants were informed that they would perform a behavioral rating task involving solid food in addition to completing questionnaires on their eating habits. They were also informed that, as the rating of solid foods may change depending on their satiety levels, they should adhere to specific breakfast suggestions on the testing day, which they received in writing and which would contain ∼250 kcal. They were then scheduled for a 1 h testing session around lunchtime (between 12:00 noon to 1:00 PM).

Upon arrival, participants completed a pretesting questionnaire asking about their hunger and thirst levels in addition to their adherence to the breakfast guidelines provided. Subsequently, the participant was given three small portions of the experimental curry dishes and asked to rate them on specific psychophysical scales. The positioning of the curry dishes was always randomized by the research chef who prepared the foods, and the researcher and participant were blind to the positioning until after the experiment. The participant was also provided with 100 ml of water to cleanse the palate between samples.

After the tasting test, the participant was moved to a different corner of the room to perform a computer-based Stroop Color and Word Test, which served as a distractor task, for 2 min. During this time, the researcher informed an assistant with a text message that the participant was ready for lunch. At the end of the Stroop Test, the assistant entered the room and offered them a lunch meal, informing them that they would need to be back in 30 min to complete a final questionnaire. The participant was then taken to a separate lounge and shown the three curries in separate large dishes (in randomized positions) and was informed that they should sample all of them and consume as much as they wanted. We encouraged to sample the three dishes without explicitly mentioning that the three meals were different and instructed them to eat until they were comfortably full. After 30 min, the participant was taken to the testing room again to complete a final questionnaire and for debriefing and remuneration. The consumed amount of each meal was covertly weighed.

#### Analysis of behavioral data

To analyze the rating data, we first performed one-way repeated-measures ANOVA with food stimulus as factor. Separate ANOVAs were performed for ratings of sweetness, thickness, oiliness, and WTP. Next, to test how ratings depended on the sugar, fat, and protein content of stimuli, and on the texture parameters CSF and viscosity, we used mixed-effects multilinear regression analysis within the GLM framework (implemented with the *fitglme* function, MATLAB) and specified the subject number as the categorical group variable to account for subject-specific effects (random effects). We adopted the global model in which we estimated both the main effects and random effects of all the relevant regressors. The response variable was the trial-by-trial rating (sweetness, thickness, oiliness, WTP). We implemented the regression shown in Tables 2 and 3. Based on previous studies (de Araujo et al., 2003; Grabenhorst et al., 2008, 2010; Zangemeister et al., 2016), we had no *a priori* hypotheses with respect to sex-based behavioral and neural effects and therefore did not perform sex-based analyses. We used Bonferroni-corrected *p* values to determine significance in these analyses, determined by the number of times a variable was used in statistical tests (e.g., sugar was used 10 times).

**Table 2.** Regression models of sweetness, thickness, and oiliness ratings

Variable	Estimate	SE	*t*	DF	*p*
Mixed effects, sweetness (AIC/BIC: 2273.3/2345.2; adjusted *R*^2^ = 0.292)					
Intercept	9.0e-05	0.0280	0.003	885	0.997
Sugar	0.436	0.0520	8.383	885	2.0e-16*
Fat	0.248	0.0383	6.473	885	1.5e-10*
Protein	0.051	0.0324	1.587	885	0.112
Mixed effects, thickness (AIC/BIC: 2012.6/2084.5; adjusted *R*^2^ = 0.478)					
Intercept	0.0001	0.024	0.005	885	0.995
Sugar	0.153	0.045	3.391	885	0.0007*
Fat	0.659	0.024	26.60	885	4.9e-115*
Protein	0.055	0.035	1.5788	885	0.114
Mixed effects, oiliness (AIC/BIC: 2357.9/2429.7; adjusted *R*^2^ = 0.227)					
Intercept	−7.3e-05	0.029	−0.002	885	0.997
Sugar	0.151	0.042	3.589	885	0.0003*
Fat	0.380	0.054	6.972	885	6.0e-12*
Protein	0.066	0.041	1.599	885	0.109
Mixed effects, thickness (AIC/BIC: 2136.9/2237.5; adjusted *R*^2^ = 0.413)					
Intercept	−8.2524	0.025	−0.00322	884	0.997
Sugar	0.1861	0.0423	4.3948	884	1.2e-05*
Protein	−0.1192	0.0500	−2.3807	884	0.017
Viscosity	−1.088	0.132	−8.2224	884	7.0e-16*
CSF	−1.4185	0.1125	−12.609	884	1.2e-33*
Mixed effects, oiliness (AIC/BIC: 2355.4/2456; adjusted *R*^2^ = 0.264)					
Intercept	−0.001	0.0283	−0.049	884	0.960
Sugar	0.1555	0.05104	3.047	884	0.002*
Protein	0.0182	0.062	−0.2904	884	0.771
Viscosity	−0.5283	0.234	−2.254	884	0.024
CSF	−0.7166	0.214	−3.338	884	0.0008*

*Significance following Bonferroni correction.

**Table 3.** Regression models of WTP bids

Variable	Estimate	SE	*t*	DF	*p*
Mixed effects, bid (AIC/BIC: 2249.9/2321.8; adjusted *R*^2^ = 0.314)					
Intercept	0.00002	0.027	0.008	885	0.993
Sugar	0.246	0.060	4.083	885	4.8e-05*
Fat	0.445	0.037	11.962	885	1.1e-30*
Protein	0.059	0.029	2.025	885	0.043
Mixed effects, bid (AIC/BIC: 2086.4/2220.5; adjusted *R*^2^ = 0.478)					
Intercept	−0.0002	0.024	−0.0122	883	0.990
Sugar	0.449	0.055	8.055	883	2.5e-15*
Fat	0.356	0.052	6.735	883	2.9e-11*
Protein	−0.142	0.055	−2.558	883	0.010
Viscosity	−0.977	0.188	−5.195	883	2.5e-07*
CSF	−0.835	0.169	−4.930	883	9.6e-07*
Mixed effects, bid (AIC/BIC: 1989.8/2061.6; adjusted *R*^2^ = 0.498)					
Intercept	2.4e-13	0.023	1.0e-11	885	1
Sweetness	0.370	0.060	6.080	885	1.6e-09*
Thickness	0.459	0.055	8.343	885	2.7e-16*
Oiliness	−0.122	0.034	−3.559	885	0.00039*
Mixed effects, bid (AIC/BIC: 2357.3/2386; adjusted *R*^2^ = 0.180)					
Intercept	−0.0001	0.030	−0.004	887	0.996
Energy	0.423	0.032	13.006	887	1.5e-35*

*Significance following Bonferroni correction.

#### fMRI data acquisition

Functional imaging data were acquired using a 3T Skyra (Siemens) scanner at the Wolfson Brain Imaging Center (WBIC, University of Cambridge). Whole-brain T2*-weighted EPIs were acquired with a repetition time of 3000 ms, echo time of 30 ms, flip angle of 90°, and 51 axial oblique slices with 3 mm isotropic resolution. Subjects were scanned in six runs. In each run, 300 volumes were acquired in an imaging time of 15 min 10 s. Importantly, each liquid food stimulus was presented 3 times in each run, to achieve a balanced design for the leave-one-run-out analysis approach described below. A high-resolution structural MPRAGE scan for normalization purposes was also acquired (voxel size, 1 × 1 × 1 mm; TR, 2300 ms; TE, 2.98 ms; inversion time, 900 ms; flip angle, 9°; total scan time, 5 min 3 s).

#### fMRI data analysis

We used the Statistical Parametric Mapping package to analyze the neuroimaging data (SPM12; Wellcome Trust Center for Neuroimaging). We discarded the first six volumes of each run to allow for magnetic saturation effects. We preprocessed the data by applying slice-timing correction, realignment, and reslicing (separately for the fMRI data from day 1 and day 2). We then segmented the data to extract white matter, gray matter, and cerebrospinal fluid and followed by coregistering the day 1 and day 2 data using the T1-weighted structural scans from each day. The data preprocessed in this way were used for multivariate voxel pattern analysis (MVPA) in individual subjects without applying normalization or spatial smoothing to the data. For univariate ROI analyses, we normalized the scans to the standard MNI coordinate system, and then applied smoothing using a Gaussian kernel with the FWHM of 6 mm. We also removed global effects from the fMRI time series using voxel-level linear model of the global signal to minimize effects correlated with global fluctuations of the signal (Macey et al., 2004).

##### MVPA

We used linear support vector machine (SVM) regression to test for encoding of continuous variables, such as the CSF, viscosity, thickness, and oiliness ratings, and the WTP ratings in local activity patterns distributed across voxels, as implemented in the Decoding Toolbox (Hebart et al., 2014). Decoding of textural parameters (CSF and viscosity) was performed with SVM regression using the CSF and viscosity value for each liquid food stimulus, whereas decoding of subjective psychophysical ratings and WTP bids was performed on the within-subject mean rating given to each food stimulus. Using a leave-one-out cross-validation method, we trained the classifier on five scan runs within each subject to predict the value of the variable of interest and then tested on the remaining run to predict the variable from distributed multivoxel activity patterns; this approach was repeated 6 times to obtain an average statistical map of *z*-transformed correlation coefficients describing the relationship between voxel activity and the variable of interest. The whole-brain MVPA was performed using a searchlight method on unsmoothed realigned data in the subject's native space. We used a linear kernel and default regularization parameter of c = 1. A 9 mm sphere was defined as a searchlight and used for MVPA, after which the searchlight moves onto a neighboring voxel. Exploratory analyses with different sphere sizes suggested that a 9 mm searchlight radius provided robust and accurate results, while small changes in sphere size did not qualitatively alter the results. We report the specific results for our main findings also with a 4 mm sphere; no additional brain areas were identified with this 4 mm sphere analysis. The resultant accuracy maps were subsequently normalized into the MNI space, following the protocol used in similar MVPA studies (Kahnt et al., 2011; Howard and Kahnt, 2017). In the second-level (group, random-effects) stage, the normalized, smoothed statistical maps resulting from the first-level MVPA were entered into one-sample *t* tests, resulting in group-level statistical parametric maps.

##### Conjunction analyses

Conjunction analyses (Nichols et al., 2005) were used to test for common encoding of different effects within the same brain region. This was achieved by testing if all of the variables of interest were significantly encoded (*p* < 0.05), in which case the null hypothesis that at least one variable was not encoded could be rejected. Although primarily used to compare shared regions in contrast files from univariate GLMs (Friston et al., 1999, 2005), conjunction analysis has also been used with accuracy maps from MVPA to indicate shared regions in taste encoding (Avery et al., 2020).

##### Finite impulse response analyses

We used a time-resolved SVR regression as previously described (Kahnt et al., 2011) to examine the encoding of our variables of interest at different time points in a trial (see Fig. 4*F*). Because we were interested not only in the activations related to the delivery of liquid food stimulus but also in possible further encoding in the period following the liquid delivery, a finite impulse response analysis was performed as implemented in SPM12, which makes no assumption about the specific time course of the HRF (Henson, 2003; Grabenhorst and Rolls, 2008). We used δ functions in the finite impulse response analysis that were spaced at intervals of 2 s and starting at the onset of the liquid delivery and spanning a period of 10 s. This basis set considers each time bin after stimulus onset individually to model the fMRI signal and can capture any possible shape of response function up to a given frequency limit. In this model, the parameter estimate for each time bin represents the average fMRI signal at that time. The activity patterns in these 2 s time bins were then used for a searchlight MVPA as described above.

##### Statistical significance testing

For all fMRI analyses, we report effects that survive correction for multiple comparisons across the whole brain using a significance level of *p* < 0.05 (family-wise error) at cluster level; this threshold was applied to statistical maps that were displayed using a cluster-forming threshold of *p* < 0.001 (uncorrected) at voxel level (Eklund et al., 2016) with cluster extent of *k* = 15 or more contiguous voxels. In addition, we used small-volume correction (*p* < 0.05, cluster-level) in structures for which we had strong *a priori* hypotheses based on previous studies, including the mid-to-lateral OFC, oral somatosensory cortex (oSSC), and ventromedial PFC. Small-volume corrections were performed in spheres of 10 mm radius for cortical areas. The spheres were centred on specific coordinates reported in previous studies as follows: mid-to-lateral OFC [32, 34, −14] and oSSC [66, −18, 12], both taken from the main effects of previous studies on oral food processing using liquid stimuli with defined sugar and fat content (Grabenhorst et al., 2010, 2014), and ventromedial PFC [−2, 40, −4] taken from a meta-analysis of value-based decision-making (Clithero and Rangel, 2014). The coordinates for small-volume corrections were determined during study design but were not preregistered.

##### Univariate ROI analysis

To ensure that statistical inference in our ROI analyses was not circular, we followed approaches used in previous studies (Behrens et al., 2008; Zangemeister et al., 2016; Rosenthal-von der Putten et al., 2019). Specifically, we used a leave-one-subject-out method in which we re-estimated a second-level analysis 22 times, each time leaving out one subject to define the ROI coordinates for the left-out subject. We then extracted the signal from the subject-specific coordinates defined in this way. Thus, the data which we used for the ROI analysis were independent from those used to define the coordinates for extracting the signal. ROIs were determined during study design but were not preregistered. Following data extraction, we applied a high-pass filter with a cut-off period of 128 s. The data were then *z*-normalized, oversampled by a factor of 10 using sinc-interpolation, and separated into trials to produce a matrix of trials against time. We generated separate matrices for each event of interest (rating trials, choice trials). We then fitted GLMs to each oversampled time point across trials separately in each subject. We fitted a GLM containing as main regressors the two key oral-texture variables CSF and viscosity. In addition, the GLMs included fat, sugar, energy content, motion parameters, and session constants as covariates of no interest. To test the statistical significance, we entered individual-subject coefficients into one-sample *t* tests (random-effects analysis, *p* < 0.05) and calculated group averages and SEs for each time point across participants, yielding the across-subject effect size time courses shown in the figures. These mean effect size time courses are shown Figure 3*B*.

##### Testing relationships between neural measures and naturalistic eating behavior

To test for relationships between neural effect sizes and behavioral variables across subjects, we extracted neural effects sizes within each individual subject form peak coordinates defined using the leave-one-subject-out procedure described above. Specifically, we extracted the neural effects sizes for encoding of CSF and viscosity from the statistical decoding maps, based on group cluster coordinates, which we defined independently by re-estimating the second-level analysis 22 times, each time leaving out one subject to define the ROI coordinates for the left-out subject. We then fitted a multilinear regression model with the neural effect sizes of OFC to CSF and viscosity as regressors to the amount of fat eaten in the *ad libitum* test as the dependent variable. To produce Figure 5*D*, we plotted the relationship between the dependent variable and its predicted values as given by the coefficients of the fitted regression model.

## Results

### Study design

To investigate the behavioral and neural mechanisms that link oral-sensory food processing to economic preferences, we tested each participant in a series of experiments (Fig. 1*A*). First, in a psychophysical test (day 1), subjects (*N* = 22) repeatedly sampled liquid food stimuli with controlled sensory and nutrient components and provided psychophysical ratings of sweetness, thickness, and oiliness. As a measure of the subjective, “economic” reward value of the foods, they also placed WTP bids to consume the foods in an incentive-compatible auction-like task (Becker et al., 1964). Second, subjects participated in two fMRI scanning sessions (days 2 and 3) in which they performed these tasks in the MRI scanner while orally sampling the liquid foods (two scanning days were required to collect sufficient data for multivoxel-pattern fMRI analyses). Liquid foods were delivered orally via food-grade tubes and computer-controlled peristaltic pumps, following established protocols (de Araujo and Rolls, 2004; Grabenhorst et al., 2010; Grabenhorst and Rolls, 2014; Zangemeister et al., 2016). Third, subjects participated in an *ad libitum* eating test (day 4) in which they made consumption choices for nutrient-controlled solid foods under naturalistic, life-like conditions. This design allowed us to investigate behavioral and neural responses to nutrient-defined foods both under controlled experimental conditions and during realistic eating behavior.

Importantly, for the psychophysical and fMRI experiments, subjects were trained to orally process the liquid foods by performing a standardized left-right tongue movement guided by a dynamic visual cue moving at a defined speed (replaced by a static cue for MRI scanning) and then hold their tongue still for several seconds before swallowing the liquids (Fig. 1*B*). This manipulation served to (1) distribute liquid foods around the oral cavity (de Araujo and Rolls, 2004; Grabenhorst et al., 2010), and (2) produce standardized oral-mechanical stimulation across stimuli and subjects, including sliding friction at a defined speed that matched our tribological measurements (see below). These factors were important given our focus on oral texture and the role of tongue movements in food-texture sensing (de Wijk et al., 2003, 2011; Koc et al., 2013).

### Design of food stimuli

We designed a set of dairy-based liquid food stimuli (“milkshakes”) with controlled nutrient and energy content to produce meaningful variation in oral-sensory stimulation, subjective sensations, and reward valuations (Table 1). We focused on sugar and fat because of their relevance for human overeating and obesity, and their role in determining sensory food qualities (Alonso-Alonso et al., 2015; Carreiro et al., 2016; Rolls, 2020; Huang et al., 2021). A 2 × 2 factorial design with sugar and fat as factors defined the following basic stimuli: (1) a LFLS liquid, (2) a HFLS liquid, (3) a LFHS liquid, and (4) a HFHS liquid. Energy content was lowest for the LFLS stimulus, intermediate for the HFLS and LFHS stimuli, and highest for the HFHS stimulus. In addition, we included (5) a LFHS and high-protein stimulus to include fat-like texture produced by protein, (6) a HFHS liquid based on soy rather than dairy milk to include both animal- and plant-based fat stimuli, and (7) a LFHS liquid that was thickened by adding carboxymethyl cellulose (a food-thickening agent used in the food industry) to produce a fat-like texture. Following previous studies (de Araujo et al., 2003; de Araujo and Rolls, 2004; Grabenhorst et al., 2008, 2010), “artificial saliva,” a largely neutral stimulus approximating the ionic composition of human saliva, was used as a rinse on each trial, and in select control analyses as described below. All stimuli except artificial saliva were vanilla-flavored. Stimuli were served at a temperature of ∼17°C that was held constant across stimuli with ice-packs.

### Measuring food texture: viscosity and CSF

We reasoned that the oral sensations produced by food textures influenced subjects' food valuations and related neural processing. Therefore, a main aim of our study was to relate subjects' behavioral and neural responses to foods to oral-texture parameters quantified with rheological and tribological food-engineering methods. Two specific texture parameters have been implicated in oral fat-sensing (Fig. 1*C*): viscosity and the CSF (Chen and Stokes, 2012; Chojnicka-Paszun et al., 2012; Stokes et al., 2013; Laguna et al., 2017).

Viscosity is a material, “bulk” food property that is measured as resistance to flow (i.e., the quotient of shear stress and shear rate) in a rotational rheometer (Fig. 1*C*); it has been shown to increase with increasing fat-droplet concentration, which is higher in fatty liquids (Bourne, 2002; de Wijk et al., 2011; Chen and Stokes, 2012; Laguna et al., 2017). By contrast, CSF is a systems property that emerges from the interaction of food with oral surfaces and is measured as the resistance to relative motion between contacting surfaces using tribological techniques (Fig. 1*C*); fatty liquids produce lower CSF, likely by coalescing fat droplets forming a load-bearing, lubricating film on oral surfaces (Dresselhuis et al., 2008b; Chojnicka-Paszun et al., 2012; Torres et al., 2018; Soltanahmadi et al., 2023). Thus, CSF and viscosity reflect physically distinct aspects of food texture (Chen and Stokes, 2012). We hypothesized that both CSF and viscosity would mediate the influence of fat content on oral sensations and food valuations, and related neural activity. Testing this hypothesis required measuring viscosity and CSF for our liquid food stimuli under biologically plausible conditions, as described next.

To approximate the friction conditions in the oral cavity, which involve the tongue sliding against the palate, we used our recently developed tribometer (Fig. 1*C*) (Huang et al., 2021) with parallel-sliding fresh pig tongues as biological surfaces that approximate the softness and surface properties of the human tongue. In using biological tissues, this approach differs from standard tribometers involving circular-rotating glass-metal surfaces. Validation tests using a series of basic fatty liquids confirmed that decreases in CSF reliably reflected increased stimulus fat content (Fig. 1*D*). In a large set of more complex liquid food stimuli that included the foods used in the present fMRI study, CSF correlated negatively with the fat content of the stimuli (*R* = −0.78, *p* = 4.6 × 10^−5^), confirming lower sliding friction for high-fat foods, whereas viscosity correlated positively with fat content (*R* = 0.83, *p* = 4.4 × 10^−6^), confirming that high-fat foods were more viscous (Fig. 1*E*). By contrast, sugar content in the stimuli showed weaker and nonsignificant correlation with CSF (*R* = −0.42, *p* = 0.063) and viscosity (*R* = 0.12, *p* = 0.59). Although CSF and viscosity themselves were correlated (*R* = −0.78, *p* = 3.4 × 10^−5^), the two parameters reflect fundamentally different aspects of food texture that become relevant during different stages of oral processing, with viscosity being a material food property and CSF resulting from food-oral surface interactions (Chen and Stokes, 2012; Stokes et al., 2013). Consistently, in the present stimulus set, CSF and viscosity discriminated between the liquid foods in different ways (Fig. 1*E*, colored stimuli): low CSF for the high-fat stimuli (HFLS, HFHS, and soy) and the fat-like carboxymethyl cellulose (CMC) differed substantially from the high CSF of the low-fat stimuli (LFLS, LFHS, and protein); in contrast, viscosity additionally distinguished the HFLS and HFHS stimuli from CMC and soy. As shown below, CSF and viscosity also had distinguishable relationships with behavioral and neural measures.

Thus, the oral-texture parameters viscosity and sliding friction tracked the fat content of the liquid foods in partly different ways and therefore likely influenced subjects' food sensations and valuations.

### Psychophysical ratings of food stimuli

**Figure 2.**
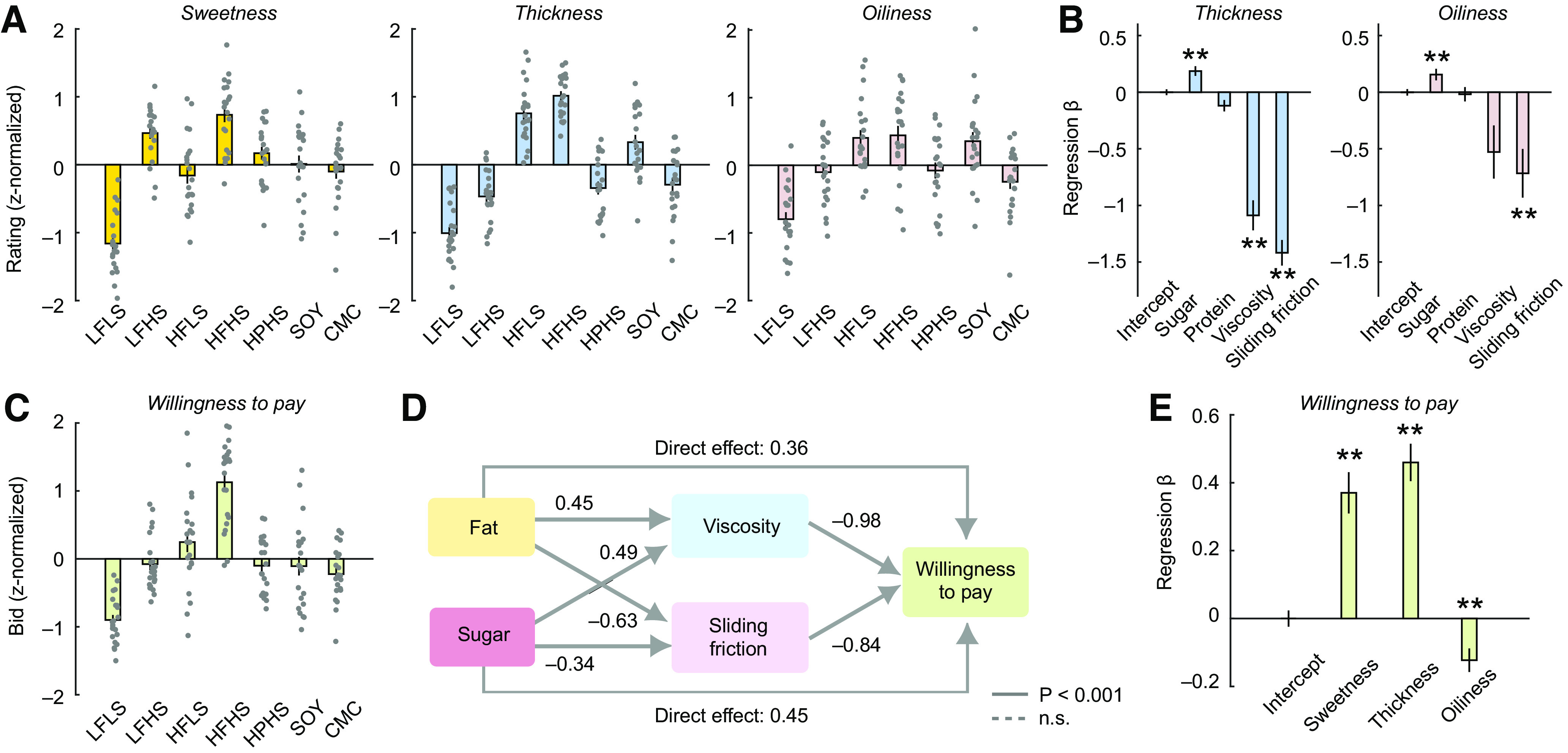
Psychophysical ratings and economic WTP bids for experimental foods. ***A***, Ratings of sweetness, thickness, and oiliness sensations for all stimuli. Error bars indicate mean ± SEM. Data points show individual subjects. HPHS, Low-fat, high-sugar, high-protein; SOY, soy-based HFHS; CMC, low-fat, high-sugar thickened with carboxymethyl cellulose. ***B***, Thickness and oiliness ratings are explained by a combination of oral-texture variables viscosity and sliding friction (mixed-effects multilinear regression, subjects as random factor). **p < 0.005, Bonferroni-corrected. ***C***, WTP bids, obtained in an incentive-compatible Becker-DeGroot-Marschak auction-like task as a measure of subjects' economic food valuations. ***D***, Mediation analysis. Path diagram describing relationships between nutrient content, texture parameters, and WTP bids (mixed-effects multilinear regression). The influence of fat and sugar content on WTP bids was decomposed into indirect effects mediated by texture variables viscosity and sliding friction and direct effects. Significance of path coefficients derived from bootstrap (1000 iterations). Protein effects were included in the model but were not significant and are not shown. ***E***, WTP depended on oral sensations of sweetness, thickness, and oiliness (mixed-effects multilinear regression; **p < 0.005).

Subjects provided psychophysical ratings on visual scales with numerical labels while sampling the liquid foods under controlled conditions and visually guided tongue movements. In their ratings, subjects discriminated the food stimuli in terms of sweetness, thickness, and oiliness (ANOVAs; sweetness: *F*_(6,147)_ = 32.81, *p* = 6.9 × 10^−25^; thickness: *F*_(6,147)_ = 63.36, *p* = 2.5 × 10^−38^; oiliness: *F*_(6,147)_ = 14.49, *p* = 6.0 × 10^−13^) (Fig. 2*A*). Notably, stimulus ratings were consistent between pretesting and scanning sessions and across the two scanning sessions (correlation across stimuli between pretest and scanning sessions: sweetness: *R* = 0.937, *p* = 0.0018; thickness: *R* = 0.978, *p* = 0.0001; correlation across stimuli between two scanning sessions: sweetness: *R* = 0.997, *p* = 9.1 × 10^−7^; thickness: *R* = 0.977, *p* = 0.0001).

**Figure 3.**
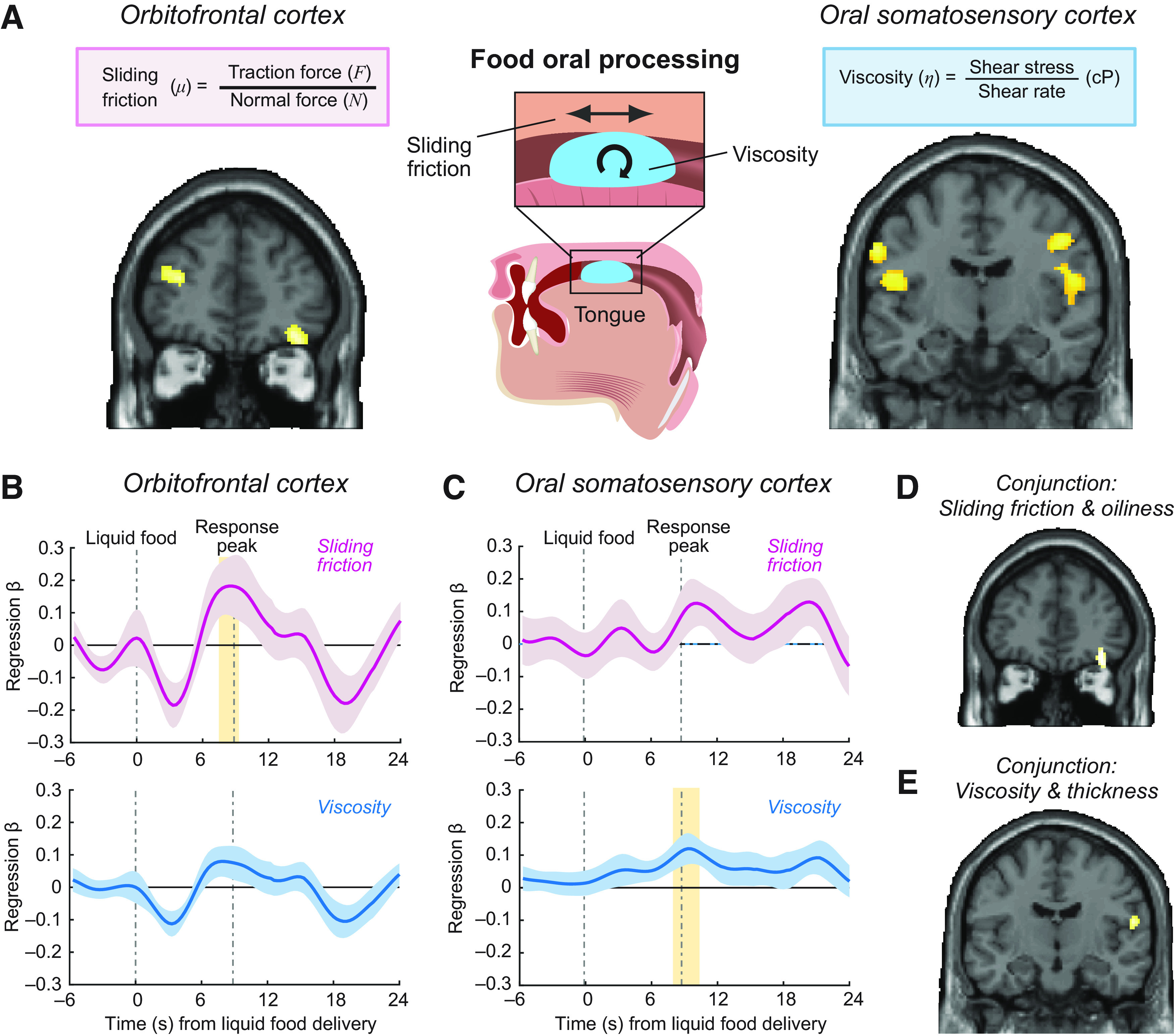
Neural encoding of specific oral-sensory food properties. ***A***, Activity pattern across voxels in OFC reflected sliding friction of liquid foods (MNI coordinates: [36, 42, −16]; *z* = 4.08; *p* = 0.012; small-volume corrected) and activity patterns across voxels in oSSC reflected viscosity (MNI coordinates: [54, −10, 22]; *z* = 4.34; *p* < 0.001, whole-brain corrected). Shown are decoding-accuracy maps derived from whole-brain searchlight analyses that used SVM regression to decode each texture variable from multivoxel activity patterns in a 9 mm sphere. Maps are shown at *p* = 0.001 with extent threshold of 15 voxels. ***B***, ROI regression of OFC cluster activity on sliding friction (*t*_(21)_ = 2.11, *p* = 0.046; one-sample *t* test across subject-specific regression betas) and viscosity (not significant). Activity timeseries was extracted from coordinates identified in a previous study. Yellow shaded region represents the period in which the regression was significant; absence of yellow shaded region represents that the regression was not significant. ***C***, ROI regression of oSSC cluster activity on viscosity (*t*_(21)_ = 2.30, *p* = 0.031; one-sample *t* test) and sliding friction (not significant). Activity timeseries was extracted from coordinates identified in a previous study. ***D***, OFC activity patterns encoded both sliding friction and oiliness in a conjunction analysis (MNI coordinates: [36, 42, −18]; *z* = 3.68; *p* = 0.002, whole-brain corrected). ***E***, oSSC activity patterns encoded both viscosity and thickness in a conjunction analysis (MNI coordinates: [58, −12, 16]; *z* = 3.76; *p* = 0.021, whole-brain corrected).

**Figure 4.**
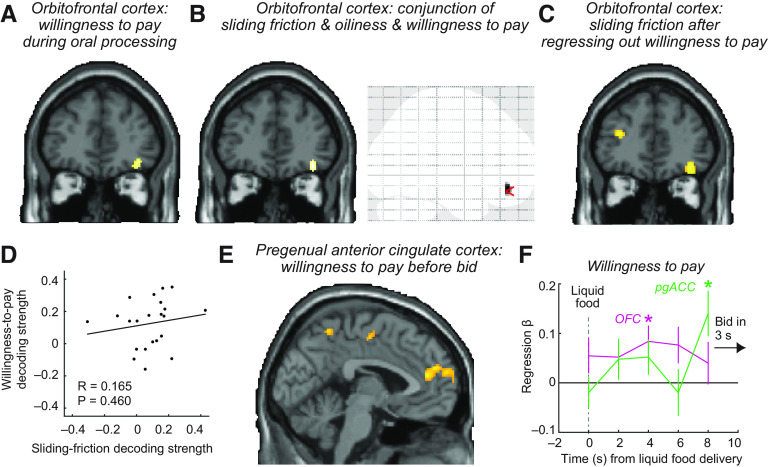
Neural encoding of economic food values and related oral-sensory properties. ***A***, Activity pattern in OFC reflected subjective, economic food reward values, measured as WTP bids during oral processing of liquid foods (MNI coordinates: [38, 40, −14]; *z* = 3.83; *p* = 0.010, small-volume corrected). Decoding-accuracy maps derived from whole-brain searchlight analysis using SVM regression to decode WTP from multivoxel activity patterns in a 9 mm sphere. Maps are shown at *p* = 0.001 with extent threshold of 15 voxels. ***B***, OFC activity patterns during oral processing encoded sliding friction, oiliness, and WTP in a three-way conjunction analysis (MNI coordinates: [36, 42, −14]; *z* = 3.74; *p* = 0.002, small-volume corrected). The OFC was the only brain area to show a significant three-way conjunction (right, transparent brain map). ***C***, OFC encoding of sliding friction remained significant when performing the decoding on CSF residuals, after regressing out variance components because of WTP bids ([36, 42, −14]; *z* = 3.99; *p* = 0.010), small-volume corrected). ***D***, Neural decoding accuracies for sliding friction and WTP in OFC were uncorrelated. ***E***, Activity patterns in pgACC encoded WTP only after oral processing before subjects placed their bids (MNI coordinates: [−2, 44, 16]; *z* = 3.73; *p* = 0.004, whole-brain corrected). ***F***, Time-resolved decoding of WTP bids in OFC and pgACC. Decoding accuracy betas obtained from a finite impulse response analysis performed in 2 s bins. The OFC β was significant in bin 3 (*t*_(21)_ = 2.60, *p* = 0.016); the pgACC β was significant in bin 5 (*t*_(21)_ = 3.05, *p* = 0.006).

**Figure 5.**
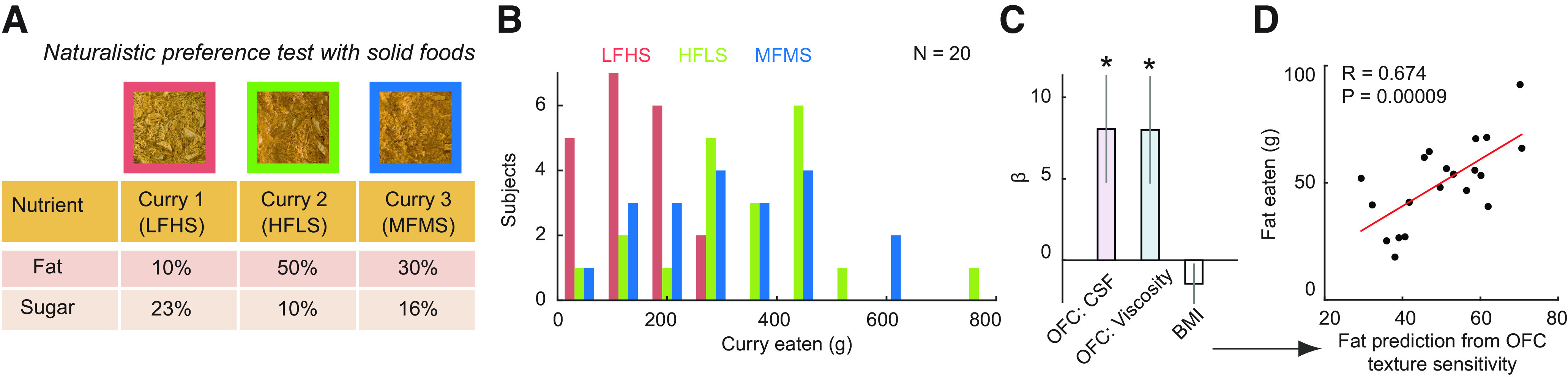
Neural encoding of oral-sensory food properties in OFC predicts preferences in a naturalistic eating test. ***A***, Design of food stimuli. Subjects could freely and repeatedly choose between three nutrient-controlled curry meals in an *ad libitum* eating test. ***B***, Variation in preference (consumed amounts) for different foods across subjects. ***C***, Modeling fat preference in the eating test from OFC neural texture sensitivity measured in the MRI scanner. We fitted a multilinear regression model with the neural sensitivity of OFC to CSF and viscosity, as well as subjects' BMI, to the amount of fat eaten in the *ad libitum* test. Neural betas were extracted from decoding-accuracy maps using independently determined OFC peak coordinates from a leave-one-subject-out cross-validation procedure (see Materials and Methods). The model provided a significant fit to the amount of fat eaten in the eating test (*F*_(3,16)_ = 4.57, full model *p* = 0.017) with both neural texture sensitivities contributing significantly (CSF regressor: *p* = 0.026; viscosity regressor: *p* = 0.027; BMI regressor: *p* = 0.264). ***D***, Relationship between the amount of fat eaten as predicted by the model based on OFC texture sensitivity and the actual amount of fat consumed by the subjects during the eating test.

Mixed-effects multilinear regressions (with subject as random factor) showed that sweetness ratings were influenced by the foods' sugar and fat content (sugar: *p* = 2.0 × 10^−16^; fat: *p* = 1.5 × 10^−10^; protein: *p* = 0.112), while thickness and oiliness ratings depended mostly on fat content (Table 2; thickness: sugar: *p* = 0.0007, fat: *p* = 4.9 × 10^−115^, protein: *p* = 0.114; oiliness: sugar: *p* = 0.0003, fat: *p* = 6.0 × 10^−12^, protein: *p* = 0.159). Thickness and oiliness ratings were of particular interest, as we expected them to reflect sensations related to oral-texture parameters, which in turn depended on fat content. We therefore substituted the fat content regressor with texture properties to test whether CSF and viscosity partly explained thickness and oiliness ratings. Both thickness and oiliness ratings were explained by combinations of the two texture parameters CSF and viscosity, in addition to a significant sugar effect (Fig. 2*B*; Table 2; thickness: sugar: *p* = 1.2 × 10^−5^, CSF: *p* = 1.2 × 10^−33^, viscosity: *p* = 7.0 × 10^−16^, protein: *p* = 0.017, oiliness: sugar: *p* = 0.0023, CSF: *p* = 0.0008, viscosity: *p* = 0.024, protein: *p* = 0.771). Specifically, higher reported thickness and oiliness were associated with lower CSF (consistent with the negative relationship between CSF and fat content, compare Fig. 1*E*). Viscosity also had a negative relationship with thickness and oiliness, which likely reflected relatively lower thickness and oiliness ratings for the non–fat-containing but viscous CMC stimulus. Indeed, when CMC was removed from the analysis, CSF coefficients remained significant and negative, but the viscosity coefficient on oiliness was no longer significant. Thickness and oiliness ratings were only partly correlated (shared variance, *R*^2^ = 0.249), indicating that they reflected partly distinct oral-texture sensations. In both thickness and oiliness regressions, the CSF regressor had higher *t* values and lower *p* values compared with viscosity (Table 2). CSF also explained more variance in both thickness and oiliness ratings: removing CSF from the regression model (with remaining regressors sugar, viscosity, protein) decreased the proportion of explained variance by 65.7% and 64.6% for thickness and oiliness ratings, respectively; removing viscosity from the model decreased the explained variance only by 34.9% and 50.0%. For comparison, removing sugar from the regression model while retaining both CSF and viscosity reduced the explained variance by 7.7% and 13.2% for thickness and oiliness ratings, respectively. Therefore, CSF was relatively more important in explaining rated oral sensations than viscosity.

Thus, the liquid food stimuli produced oral sensations that reflected the foods' nutrient composition and related texture qualities, with both CSF and viscosity contributing to sensations of oiliness and thickness. We next examined how these food properties and related oral sensations influenced subjective reward valuations, as measured by subjects' WTP to consume the foods.

### Subjective reward valuations of foods depend on texture parameters and oral sensations

Subjects performed an incentive-compatible auction-like task (Becker et al., 1964) in which they placed WTP bids on each of the liquid foods to win the opportunity to consume 250 ml of the offered liquid after the testing session. Bids were consequential, in that subjects were informed that the bid from a randomly chosen trial would be implemented. WTP bids varied significantly across food stimuli (Fig. 2*C*, left; ANOVA: *F*_(6,147)_ = 30.3, *p* = 1.7 × 10^−23^). On average, subjects placed the highest bids on the HFHS stimulus, which was highest in sugar, fat, and energy content (*p* < 0.001, Bonferroni-corrected *post hoc t* tests). Despite this general effect, there were also considerable interindividual differences in food valuation (Fig. 2*C*, gray data points), indicating that WTP bids reflected individual preferences. Notably, bids were consistent between pretesting and scanning and across scanning sessions (correlation between pretesting and scanning sessions: *R* = 0.976, *p* = 0.0001; correlation between scanning sessions: *R* = 0.967, *p* = 0.0003).

Mixed-effects regression confirmed that higher bids were associated with higher fat and sugar content of the food stimuli (Table 3; sugar: *p* = 4.8 × 10^−5^; fat: *p* = 1.2 × 10^−30^); effects of protein were limited by design (*p* = 0.043). A previous study found a fat–carbohydrate interaction effect on WTP-measured valuation in response to visually cued foods (DiFeliceantonio et al., 2018). We tested for a fat–sugar interaction effect on WTP in an additional analysis, but this interaction was not significant (*p* = 0.159, mixed-effect regression) while fat and sugar main effects remained significant (both *p* < 1.0 × 10^−5^).

We tested the effects of oral-texture parameters on WTP bids in a mediation analysis, using Structural Equation Modeling implemented by a series of mixed-effects regression models. This approach estimated both direct effects of fat and sugar on WTP and indirect effects that were mediated by the oral-texture variables CSF and viscosity. Regression-derived path coefficients (Fig. 2*D*) showed that CSF and viscosity had significant negative effects on WTP (CSF: *p* = 9.6 × 10^−7^; viscosity: *p* = 2.5 × 10^−7^), showing that subjects placed higher bids on foods that produced low sliding friction and that were less viscous. Direct effects of fat and sugar were significantly reduced by the inclusion of CSF and viscosity in the regression, although they remained significant (sugar: *p* = 2.5 × 10^−15^; fat: *p* = 2.9 × 10^−11^). Thus, CSF and viscosity partially mediated the effects of fat and sugar on WTP bids.

Notably, as for the oiliness and thickness ratings, CSF seemed relatively more important in explaining variance in WTP bids: removing CSF from the regression model (with remaining regressors sugar, viscosity, protein) reduced the proportion of explained variance by 72.6%, whereas removing viscosity reduced the explained variance by 58.7%. For comparison, removing sugar from the regression model while retaining both CSF and viscosity reduced the explained variance by 34.7%. Model comparisons showed that a simpler model based on the energy content of the food stimuli was insufficient for explaining WTP bids (AIC and BIC favored more complex models that included fat and sugar as well as oral-texture variables, Table 3).

Finally, we examined how WTP bids were related to psychophysically reported oral sensations using sweetness, thickness, and oiliness ratings as regressors. Both sweetness and thickness had significant positive influences on bids (sweetness: *p* = 1.6 × 10^−9^; thickness: *p* = 2.7 × 10^−16^); oiliness had a significant negative influence on bids (*p* = 0.00003) and therefore seemed to partially capture the negative effect of sliding friction on WTP bids reported above (Fig. 2*E*). The behavioral analyses described above were designed to test specific hypotheses regarding the relationships between particular variables. For descriptive purposes, we also report the full cross-correlation matrix between these variables (Table 4).

**Table 4.** Cross-correlation matrix of food components, psychophysical ratings, and WTP across all liquid food stimuli (mean Pearson correlation coefficients across participants)

	Fat	Sugar	Protein	Viscosity	CSF	Energy	Sweetness	Thickness	Oiliness	WTP
Fat	1	—	—	—	—	—	—	—	—	—
Sugar	−0.12	1	—	—	—	—	—	—	—	—
Protein	−0.09	0.23	1	—	—	—	—	—	—	—
Viscosity	0.41	0.36	−0.25	1	—	—	—	—	—	—
CSF	−0.60	−0.22	0.12	−0.91	1	—	—	—	—	—
Energy	0.83	0.12	0.45	0.30	−0.51	1	—	—	—	—
Sweetness	0.24	0.57	0.18	0.13	−0.21	0.37	1	—	—	—
Thickness	0.79	0.10	0.03	0.40	−0.60	0.74	0.49	1	—	—
Oiliness	0.49	0.20	0.11	0.26	−0.37	0.52	0.35	0.60	1	—
WTP	0.50	0.29	0.10	0.18	−0.35	0.54	0.67	0.73	0.38	1

Thus, subjective food valuations depended on the sugar and fat content of the stimuli. The effect of fat on valuations was partly mediated by the oral-texture parameters CSF and viscosity, and their subjective correlates oiliness and thickness. We next examined how oral-texture parameters and food valuations were related to neural activity patterns in different brain regions.

### Neural encoding of food texture: sliding friction and viscosity

We used fMRI to measure neural responses when subjects orally processed the liquid foods and applied multivariate analysis approaches to identify neural encoding of our key oral-texture variables. Specifically, we used a whole-brain searchlight approach that decoded CSF and viscosity from local activity patterns (fMRI signal intensities in a local group of voxels) while the liquid food was in the subjects' mouth on each trial; the searchlight approach performed this decoding across the whole brain by moving a sphere of 9 mm radius that defined the voxels in the decoding sample systematically through the brain. To do so, we trained a SVM regression algorithm to decode oral-texture variables and subjective stimulus ratings from the fMRI signal intensity patterns distributed across a 9 mm sphere that defined the voxels for the current decoding sample; the sphere was systematically moved across the whole brain to produce decoding accuracies for each brain area (Hebart et al., 2014; Haynes, 2015). We performed cross-validation by systematically training the classifier on data from all but one scanning runs to test performance on the remaining run (leave-one-run-out).

Cross-validated decoding revealed that activity patterns in a region of the mid-to-lateral OFC reflected our key variable CSF (Fig. 3*A*; MNI coordinates: [36, 42, −16]; *z* = 4.08; *p* = 0.012; small-volume corrected based on coordinates taken from a previous study (Grabenhorst et al., 2010) (Table 5). This finding implied that during the oral processing of liquid foods, OFC activity patterns reflected the sliding friction produced by the interaction of a specific liquid food with oral surfaces (with high-fat foods producing lower sliding friction, see Fig. 1). In addition, CSF could be decoded from a region of the postcentral gyrus consistent with the location of the oSSC (Grabenhorst and Rolls, 2014), although this effect was weaker than in OFC (MNI coordinates: [58, −14, 14]; *z* = 3.20; *p* = 0.044; small-volume corrected based on coordinates from a previous study (Grabenhorst and Rolls, 2014) (Table 5). We found that overlapping areas of OFC and oSSC showed significant relationships to the material food property viscosity (Fig. 3*B*, Table 5; OFC: MNI coordinates: [40, 38, −12]; *z* = 4.72; *p* < 0.001, whole-brain corrected; oSSC: MNI coordinates: [54, −10, 22]; *z* = 4.34; *p* < 0.001, whole-brain corrected). Thus, activity patterns in OFC and oSSC also reflected the viscosity of the liquid foods, which differed based on the foods' fat content (with high-fat foods being more viscous, see Fig. 1). These results were confirmed when decoding was performed with a smaller sphere of 4 mm radius (CSF, OFC: *z* = 4.17, *p* = 0.042; CSF, oSSC: *z* = 3.39; *p* = 0.048; viscosity, OFC: *z* = 4.02; *p* = 0.030; viscosity, oSSC: *z* = 4.48; *p* = 0.034). The results were also not explained by energy content: in control analyses, decoding based on CSF and viscosity residuals after regressing out energy showed significant effects in OFC and oSSC (CSF, OFC: *z* = 4.75, *p* = 0.045; CSF, oSSC: *z* = 3.94; *p* = 0.005; viscosity, OFC: *z* = 4.92; *p* < 0.0001; viscosity, oSSC: *z* = 4.23; *p* < 0.0001). Decoding on CSF residuals after removing shared variance with viscosity (and vice versa) was unsuccessful (in either direction), indicating that regressing out shared variance removed important information. We performed control analyses by repeating the decoding procedure for CSF and viscosity with a delayed onset time that captured neural activity measured during the rinse period on the same trials, after subjects had swallowed the liquid foods. No significant effects were found in the rinse period even at a more lenient threshold (*p* < 0.005, uncorrected), which confirmed that neural encoding of CSF and viscosity was specific to the time when the liquid food was in the mouth, and that there was no lingering stimulus (e.g., because of residual mouthcoating) that could have enabled texture decoding from subsequent neural activity.

**Table 5.** fMRI results*^a^*

Effect	Brain area	*x*	*y*	*z*	Cluster size	*Z*	*p*
CSF	OFC	36	42	−16	30	4.08	0.012 svc
	oSSC	58	−14	14	53	3.46	0.044 svc
	Occipital	40	−74	32	640	4.15	0.000
	Occipital	−16	76	36	211	4.05	0.041
Viscosity	OFC	50	44	−4	1444	4.72	0.000
	oSSC	54	−10	22	3663	4.34	0.000
	oSSC	−54	−10	20	2169	3.39	0.000
	Medial prefrontal	20	62	14	371	3.70	0.020
	Cerebellum	6	−70	−46	1188	4.54	0.000
	Cerebellum	24	−70	−16	896	4.24	0.000
	Cerebellum	−28	−72	−32	561	3.94	0.003
	Occipital	32	−78	22	2779	4.51	0.000
Oiliness	OFC	34	40	−8	57	3.58	0.005 svc
Thickness	oSSC	60	−14	18	31	3.54	0.012 svc
WTP	OFC	38	40	−14	34	3.83	0.010 svc
	Posterior cingulate	12	−52	22	225	3.85	0.029
WTP delayed	pgACC	−2	52	24	352	3.77	0.004
	Anterior insula	36	4	8	361	4.22	0.004
	Posterior insula	−54	−18	2	213	4.25	0.041
	Cerebellum	18	−54	−26	376	4.25	0.003
	Parietal	−42	−10	46	438	4.19	0.001
	Parietal	42	−12	36	496	4.13	0.001
WTP residuals, after regressing out CSF	OFC	38	40	−14	35	3.91	0.026 svc
CSF residuals, after regressing out WTP	OFC	36	42	−14	90	3.99	0.010 svc
Conjunction: viscosity & thickness	oSSC	58	−12	16	59	3.76	0.021
Conjunction: CSF & oiliness	OFC	36	42	−18	84	3.68	0.002
Conjunction: CSF & oiliness & WTP	OFC	38	40	−16	10	3.41	0.017 svc
Conjunction: CSF & oiliness & WTP delayed	—	—	—	**—**	—	—	—
CSF during rinse	—	—	—	—	—	—	—
Viscosity during rinse	—	—	—	—	—	—	—

*^a^*svc, Small-volume correction on predefined coordinates (see Materials and Methods).

**Table 6.** Nutrient and caloric composition of the foods in the *ad libitum* eating test*^a^*

	HFLS	MFMS	LFHS
Energy	182	137	102
Fat	10	4.5	1.5
of which saturates	1	0.6	0.8
Carbohydrate	19	20	18
of which sugars	6.1	7.1	7.2
Fiber	1.7	1.7	2.1
Protein	3.4	3.4	3
Salt	0.71	0.69	0.29

*^a^*Energy: kcal/100 g; fat, carbohydrate, fiber, protein, salt: g/100 g.

To further distinguish between the neural encoding of CSF and viscosity in OFC and oSSC, we used ROI analyses by extracting the cluster-based fMRI signal from predefined coordinates of OFC (Grabenhorst et al., 2010) and oSSC (Grabenhorst and Rolls, 2014). We then performed time-resolved multilinear regression analysis on the extracted neural signals in which both CSF and viscosity were entered as covariates and thus competed to explain variance in neural activity (other covariates were fat, sugar, and energy content; see Materials and Methods). This analysis indicated that activity in the identified OFC and oSSC clusters contributed in different ways to the encoding of oral texture: cluster-based OFC activity showed a significant relationship to CSF but not viscosity (Fig. 3*B*, CSF: *t*_(21)_ = 2.11; *p* = 0.046; one-sample *t* test across subject-specific regression betas), whereas cluster-based oSSC activity showed the opposite pattern of a significant relationship to viscosity but not CSF (Fig. 3*C*, viscosity: *t*_(21)_ = 2.30; *p* = 0.031). Although this result does not demonstrate a formal double dissociation, it shows that CSF and viscosity contributed differently to explaining OFC and oSSC activity when these variables competed in the same regression model. Simple fat and sugar effects were not significant in both OFC and oSSC. An additional analysis which included only fat and sugar regressors showed no significant effect in OFC and a significant sugar effect in oSSC (*t*_(21)_ = 2.65, *p* = 0.015), indicating that objective fat and sugar content per se did not explain OFC and oSSC activity well. The observed partial neural separation of CSF and viscosity is consistent with concepts from the food-engineering literature whereby distinct aspects of oral food processing are thought to reflect the bulk (mass-related) food property viscosity and the thin-film (lubrication-related) property CSF (Chen and Stokes, 2012; Stokes et al., 2013; Shewan et al., 2020).

Next, we trained the SVM regression algorithm to decode subjective food ratings related to oral-texture variables from neural activity patterns. Multivariate decoding of oiliness and thickness ratings given to the different food stimuli showed that OFC activity patterns reflected subjective oiliness (Table 5; MNI coordinates: [34, 40, −8]; *z* = 3.58; *p* = 0.005, small-volume corrected), whereas oSSC activity patterns reflected subjective thickness (MNI coordinates: [60, −14, 18]; *z* = 3.54; *p* = 0.012, small-volume corrected). We performed conjunction analyses (Nichols et al., 2005) between the statistical decoding-accuracy maps for texture variables and ratings to test whether the same areas that encoded texture variables also encoded subjective oral sensations. Conjunction analyses showed that activity patterns in the mid-to-lateral OFC area encoded both CSF and subjective oiliness (Fig. 3*D*; MNI coordinates: [36, 42, −18]; *z* = 3.68; *p* = 0.002, whole-brain corrected), whereas oSSC activity patterns reflected both viscosity and subjective thickness (Fig. 3*E*; MNI coordinates: [58, −12, 16]; *z* = 3.76; *p* = 0.021, whole-brain corrected).

Thus, OFC and oSSC encoded partly distinct food-texture properties and related sensations during the oral processing of liquid food stimuli: OFC was particularly sensitive to the sliding friction produced by moving the liquid foods between the oral surfaces, whereas oSSC was particularly sensitive to the foods' viscosity. Given that these texture variables and related oral sensations contributed to subjective reward valuations (Fig. 2), we next investigated neural activity in relation to subjects' WTP bids.

### Neural encoding of subjective food valuations

Multivariate decoding using SVM regression showed that activity patterns in the mid-to-lateral OFC region during oral food processing reflected subjects' economic food valuations, as measured by WTP bids (Fig. 4*A*, Table 5; MNI coordinates: [38, 40, −14]; *z* = 3.83; *p* = 0.010, small-volume corrected; decoding with 4 mm radius sphere: *z* = 3.89; *p* = 0.023). OFC activity therefore not only reflected the physical food property of sliding friction but also individuals' subjective food valuations, which depended on sliding friction. In a three-way conjunction analysis between CSF, oiliness, and WTP, the OFC was the only brain area to significantly encode all three variables (Fig. 4*B*, MNI coordinates: [36, 42, −14]; *z* = 3.74; *p* = 0.002, small-volume corrected). Thus, neural representations of CSF, oiliness, and food valuations overlapped in OFC.

We performed strict tests of whether the encoding of sliding friction in OFC was separate from its encoding of value, to rule out that the conjunction result described above was entirely driven by shared variance components between WTP bids and CSF. In these tests, we first regressed out the variance in CSF that was related to WTP bids in each subject, and then repeated the multivariate decoding on the CSF residuals. Decoding with these CSF residuals after correcting for WTP showed a significant effect of sliding friction in OFC (Fig. 4*C*; [36, 42, −14]; *z* = 3.99; *p* = 0.010, small-volume corrected). In a converse analysis, decoding with individual subjects' WTP bids after regressing out CSF also revealed a significant effect of WTP bids in OFC ([34, 46, −18]; *z* = 3.91; *p* = 0.026). Thus, both CSF and WTP bids explained distinct variance portions in OFC activity patterns. Further, neural decoding accuracies for CSF and WTP bids (extracted from predefined OFC coordinates: [32, 34, −14]) (Grabenhorst et al., 2010) were uncorrelated (Fig. 4*D*), indicating partly independent representations. Consistently, a model comparison on the univariate OFC signal (compare Fig. 3*B*) showed that a model based on subjective variables (sweetness, thickness, oiliness, WTP) did not provide a significantly better fit than a model based on objective variables (sugar, fat, viscosity, CSF, energy; *t* test on AIC, *t*_(20)_ = 1.76, *p* = 0.093). The same results were found for the univariate oSSC signal (*t*_(20)_ = 1.57, *p* = 0.131). Thus, the OFC appeared to be a key integration site during oral food processing that combined distinct information about physical food-texture properties and subjective food reward valuations.

Different from the OFC, encoding of WTP bids during oral processing was not found in the oSSC, which therefore encoded viscosity and thickness as shown above but not value. We also did not find evidence for WTP encoding during oral processing in the medial PFC, including the ventromedial PFC, which was previously shown to encode WTP for visually cued foods (Plassmann et al., 2007; Lebreton et al., 2009; Suzuki et al., 2017; DiFeliceantonio et al., 2018). Based on these previous studies, we reasoned that medial prefrontal activity might encode subjective value at a later trial stage when subjects were asked to report their bids in response to visual cues. Testing for encoding of WTP with a delayed onset time of seven seconds (just after the tasting period and before the rating period), we found a strong effect related to WTP in the pregenual anterior cingulate cortex (pgACC) (Fig. 4*E*, MNI coordinates: [−2, 44, 16]; *z* = 3.73; *p* = 0.004, whole-brain corrected; decoding with 4 mm radius sphere: *z* = 3.36; *p* = 0.027 at lower threshold of *p* = 0.005 uncorrected), a region that partly overlaps with ventromedial PFC and has previously been implicated in food valuation (Grabenhorst et al., 2010). The pgACC cluster extended into adjacent ventromedial PFC with a subpeak at [−10, 44, 4]. A time-resolved decoding analysis (see Materials and Methods) further confirmed that OFC encoded subjective value primarily during food oral processing, whereas pgACC encoded subjective value only after oral processing, before subjects were required to report their food evaluations (Fig. 4*F*). A conjunction analysis showed that neither OFC nor pgACC jointly encoded the oral-texture variables CSF and viscosity alongside WTP bids in this later period following oral processing.

Thus, during oral processing, activity patterns in OFC reflected subjects' food reward valuations alongside oral-texture variables. This initial integration of texture parameters with subjective values in the OFC during oral processing was followed by a “pure” value signal in pgACC shortly before subjects reported their valuations.

### Naturalistic food-preference test and relationship to neural measures

We next asked whether the neural texture sensitivity of the OFC, measured during controlled experimental conditions in the MRI scanner, was related to subjects' food preferences under life-like, naturalistic conditions. The same subjects that participated in MRI scanning (*N* = 20) also participated in a three choice *ad libitum* eating test during which they freely and repeatedly chose between three solid foods, offered in different serving trays, to consume their lunch. The design of the foods and testing conditions followed a previous study which demonstrated differences in fat preference between healthy participants and participants with obesity related to mutations in the Melanocortin-4-receptor (van der Klaauw et al., 2016). The foods were matched in flavor (a vegetarian version of chicken korma) and visual appearance (van der Klaauw et al., 2016) but differed in fat and sugar composition and included a low-fat, high-sugar stimulus, a HFLS stimulus, and a mid-fat mid-sugar (MFMS) stimulus (Fig. 5*A*; Table 6). Before the *ad libitum* eating test, subjects sampled and rated a small quantity of each of the three curry foods (performed in a separate room under conditions designed to prevent carryover effects between ratings and the eating test). Ratings suggested that subjects discriminated the foods in terms of sweetness, oiliness, savoriness, and pleasantness (ANOVAs with *post hoc t* tests corrected for multiple comparisons, all *p* < 0.001), but not in terms of thickness and saltiness (*p* > 0.075).

During the *ad libitum* eating test, the offered foods elicited substantial interindividual variation in how much subjects consumed of each of the foods (Fig. 5*B*, ANOVA *p* < 1.8 × 10^−5^). On average, subjects consumed more of the HFLS and MFMS foods compared with the LFHS food (both *p* < 0.0001, *t* tests, Bonferroni-corrected). As our study focused on preferences for fat, the key variable of interest was the amount of fat consumed in the eating test. If oral texture sensations contributed to subjects' fat preferences, then neural sensitivity to texture variables in the OFC might be related to subjects' eating phenotypes measured under naturalistic conditions. To test this hypothesis, we fitted a multilinear regression model with the neural sensitivity of OFC to CSF and viscosity as regressors to the amount of fat eaten in the *ad libitum* test. Neural betas were extracted from decoding-accuracy maps using independently determined OFC peak coordinates from a leave-one-subject-out cross-validation procedure (see Materials and Methods).

Consistent with our prediction, neural sensitivity of OFC to CSF and viscosity explained individual differences in eating phenotypes: subjects whose OFC encoded CSF and viscosity more strongly in the scanner subsequently consumed more fat in the life-like eating test (Fig. 5*C*,*D*; *F*_(3,16)_ = 4.57, full model: *p* = 0.017; CSF regressor: *p* = 0.026; viscosity regressor: *p* = 0.027). Similar results were found in single linear regressions (CSF: *p* = 0.046, viscosity: *p* = 0.031) and in a random-effects regression that treated subject as random factor (CSF: *p* = 3.3 × 10^−31^, viscosity: *p* = 0.046). The regression model based on neural sensitivity to oral texture explained a substantial portion of the variance in consumed fat between subjects (*R*^2^ = 0.461). The effect of neural texture sensitivity on fat preference was independent of subjects' BMI, which we included as a control covariate (BMI regressor: *p* = 0.264). In a model that also included sex and age covariates, the CSF regressor remained significant (*p* = 0.0028) but not the viscosity regressor (*p* = 0.081) with no effect of sex (*p* = 0.515) but a significant effect of age (*p* = 0.022). As a further control, no significant relationship was found between neural texture sensitivity and the total amount of food eaten (*F*_(3,16)_ = 2.63, full model: *p* = 0.0855; CSF regressor: *p* = 0.122; viscosity regressor: *p* = 0.0521; BMI regressor: *p* = 0.499), indicating that the effect was specific to fat preference. To further test the robustness of the identified predictive relationship, we performed an out-of-sample prediction by predicting each subject's consumed amount of fat based on a neural texture model that did not include the predicted subject's data (leave-one-subject-out resampling). The model remained significant (*p* < 0.05) in 19 of 20 tests with the proportion of explained variance in fat consumption ranging from *R*^2^ = 0.377 to *R*^2^ = 0.595 (mean ± SEM, *R*^2^ = 0.466 ± 0.01). We also tested for relationships between fat consumption and neural texture sensitivity of oSSC, which encoded oral-texture variables but, unlike OFC, did not encode subjective reward valuations. Different from OFC, texture sensitivity of oSSC was not related to fat consumption in the eating test (*F*_(3,16)_ = 0.556, full model: *p* = 0.649; CSF regressor: *p* = 0.424; viscosity regressor: *p* = 0.622; BMI regressor: *p* = 0.670). As described in the Introduction, CSF and viscosity reflect related but mechanically and conceptually distinct oral-texture parameters; accordingly, we included both variables in the analysis above. For completeness, we also compared models that regressed the amount of fat eaten in the *ad libitum* eating test on OFC sensitivity to both CSF and viscosity, only CSF, or only viscosity. Akaike Information Criterion (AIC) and Bayesian Information Criterion (BIC) showed lowest values for the model based on both CSF and viscosity (AIC/BIC: −154.93/−151.05) compared with models based only on CSF (AIC/BIC: −151.96/−149.97) or only on viscosity (AIC/BIC: −152.54/−150.55).

Thus, neural sensitivity of the OFC to oral-texture parameters, measured under controlled experimental conditions, explained individual differences in subjects' preference for high-fat foods, measured on a separate testing day during naturalistic conditions.

## Discussion

We measured behavioral and neural responses when healthy volunteers consumed liquid foods that varied in nutrient (fat, sugar) composition and sensory, oral-texture qualities. Our main finding is that a region of the human OFC responded to foods in the mouth by encoding the CSF, a mechanical oral-texture parameter that resulted from interactions between liquid foods and oral surfaces.

Specifically, OFC activity patterns reflected the low sliding friction and oiliness sensations produced by high-fat foods, as well as the subjective economic valuations that depended on sliding friction. The neural encoding of sliding friction in OFC was partly independent from its encoding of economic value, suggesting separate but overlapping neural representations. These effects were not explained by viscosity, a distinct texture parameter that reflects material food properties rather than food-oral surface interactions and separately influenced value and neural activity. The neural integration of food-texture parameters with economic value was specific to OFC and not found in oral SSC or pgACC, which separately encoded food textures and value, respectively. Importantly, oral-texture sensitivity of OFC predicted the amount of fat that subjects consumed in a naturalistic eating test. These results identify the human OFC as a key brain structure that detects oral fat from sliding friction and translates this sensory information into economics values that guide food preferences.

The conditions under which CSF was measured and related to behavior and neural activity are distinctive features of our study. Using tribological techniques that approximated oral surface conditions with pig tongues (Huang et al., 2021), we showed that the CSF reliably indicated the fat content of liquid foods. The physical mechanism by which fatty liquids lower oral sliding friction involves coalescence of fat droplets on oral surfaces which form an adhering fat layer that likely influences oral sensation (Dresselhuis et al., 2008b; Chojnicka-Paszun et al., 2012; Shewan et al., 2020). Our approach followed earlier studies that used pig tongues to link food fat content to CSF (Dresselhuis et al., 2008a; Huang et al., 2021) and applied this method for the first time to human fMRI and behavioral measures of economic food valuations. To match oral processing and tribological testing conditions, we trained subjects to perform controlled tongue movements with a speed approximating the measuring conditions of our tribometer.

Subjects' behavioral responses to the foods confirmed that CSF contributed to sensations of oiliness and thickness, and partly mediated the influence of fat on subjective valuations. Previous studies linked human food ratings to oral-texture attributes (Kokini, 1987; Kadohisa et al., 2005; Laguna et al., 2017) but did not study CSF valuations with incentive compatible economic auctions. We found that lower sliding friction typically elicited higher valuations, indicating that subjects placed higher bids for the opportunity to consume foods with a smooth, oily texture typical of high-fat stimuli. These oral-texture effects were not accounted for by sugar, sweetness, and caloric content, which were regression covariates. While our mediation model linked CSF and viscosity directly to subjective WTP bids, a further refinement could model subjective fat and sugar perception as an additional, intermediate step. Different from a previous study using visual food cues (DiFeliceantonio et al., 2018), we found no fat–sugar interaction effect on valuation, which may be because of our use of oral food stimuli or our specific stimulus set.

In previous fMRI studies, fat-containing and texture-controlled foods activated OFC, oSSC, and pgACC (de Araujo and Rolls, 2004; Grabenhorst et al., 2010; Eldeghaidy et al., 2011; Stice et al., 2011; Grabenhorst and Rolls, 2014; Eldeghaidy et al., 2016; Zangemeister et al., 2016). We extended these approaches by combining (1) novel food-engineering methods that quantified oral-texture parameters on biological tissues, (2) incentive-compatible measures of economic value, and (3) a naturalistic food-preference test. This approach allowed us to uncover a functionally and anatomically specific role of the human mid-to-lateral OFC in oral food processing. This OFC region parametrically encoded sliding friction shown using both multivoxel-pattern decoding, which reflects local distributed voxel-activity patterns, and univariate multilinear regression, which reflects the dominant cluster signal of these voxels (Haynes et al., 2007). Although oSSC also encoded oral texture and pgACC encoded economic value, the OFC was the only area in which these variables were jointly encoded. Previous studies identified roles of the insula in processing food attributes evoked either by oral stimulation or visual food pictures, including at 7 Tesla MRI field strengths (Avery et al., 2020, 2021). Our findings suggest that food texture and related value evoked by oral food stimuli are primarily represented in OFC and oSSC, although future studies could investigate possible insula contributions with targeted approaches.

The OFC has long been implicated in food processing and related functions in reward and emotion (Rolls et al., 1999, 2003; Tremblay and Schultz, 1999; Rolls and Grabenhorst, 2008; Grabenhorst et al., 2010; Grabenhorst and Rolls, 2011; Howard and Kahnt, 2017). It receives direct inputs from oSSC (Carmichael and Price, 1995) and anterior insular taste cortex (Baylis et al., 1995). The same region of human mid-to-lateral OFC was previously implicated in oral-fat processing (Grabenhorst et al., 2010; Grabenhorst and Rolls, 2014), but the underlying oral-mechanical parameters and their relation to economic value and eating behavior remained unclear. Single-neuron recordings in macaque OFC demonstrated detailed representations of sensory food properties (Rolls et al., 1999, 2003; Verhagen et al., 2003; Kadohisa et al., 2005), including neurons that encoded either CSF or viscosity, which were intermingled in OFC (Rolls et al., 2018). Although the spatiotemporal resolution of fMRI cannot recover such detailed representations (Logothetis, 2008), our multivoxel decoding results showed that human OFC was sensitive to both CSF and viscosity. Fully dissociating CSF and viscosity in a decoding analysis was not possible; nevertheless, the univariate fMRI signal in OFC was dominated by CSF rather than viscosity, indicating a robust sliding-friction representation in the human OFC (although a formal double dissociation was not demonstrated). We note that cleanly dissociating CSF and viscosity is a current unresolved challenge in the food-engineering field.

The present results inform our understanding of neural reward mechanisms that contribute to overconsumption of high-fat foods and obesity. We found that the sensitivity with which the OFC encoded the oral-texture variables CSF and viscosity predicted subjects' fat preferences in a naturalistic eating test. This finding is consistent with studies that linked structure and function of the human OFC to eating behavior (Morys et al., 2022). For example, abnormal eating in frontotemporal dementia has been associated with OFC pathology (Woolley et al., 2007), variation in OFC gray-matter volume has been linked to obesity (Shott et al., 2015; Hall et al., 2023), and resting-state OFC functional connectivity is associated with liking for sweet foods and BMI (Rolls et al., 2023). Our findings suggest a possible mechanism by which the OFC could contribute to eating behavior: the OFC may translate information about the “objective,” material properties of foods, including their nutrient content and oral texture, into subjective sensations and economic values that guide eating behavior. The mapping from nutrients and sensory food properties to subjective values likely depends on individual-specific synaptic weights stored on OFC neurons (Rolls and Grabenhorst, 2008). Indeed, our behavioral results showed that oral-texture parameters partly mediated the effect of fat on individuals' subjective valuations, matching our recent findings in macaques (Huang et al., 2021). We note that the relationship between fat preferences and OFC encoding of oral texture was found in a relatively small sample of *N* = 20 participants. Although convergent evidence points to a key role of the OFC in food-intake control (reviewed above) and our result was robust to several control analyses, future studies should replicate this finding in a larger sample.

Our findings have implications for a central question in decision neuroscience: how are subjective values neurally constructed from sensory inputs? Suzuki et al. (2017) showed that multivoxel activity in lateral OFC in response to visual food cues reflected nutrient components and both medial and lateral OFC regions reflected economic value. Other studies found that values evoked by visual food cues were encoded in ventromedial PFC (Lebreton et al., 2009; Tang et al., 2014). Here, by examining oral food processing, we provide evidence that subjective value may be initially constructed from oral-sensory inputs in the mid-to-lateral OFC before being encoded as sensory-independent value in pgACC. Specifically, whereas OFC activity encoded WTP while subjects orally sampled liquid foods, pgACC activity encoded WTP only after subjects had swallowed the foods and prepared to report their valuations. Notably, only OFC but not pgACC jointly represented value and oral-texture signals and oiliness sensations on which valuations were based. Thus, our findings point to a key role of the mid-to-lateral OFC in constructing food value from oral-sensory inputs.

Our findings have implications for food engineering and food design. Advances in food science identified sliding friction as a key factor in the design and acceptability of food (Chen and Stokes, 2012; Wang and Chen, 2017; Upadhyay et al., 2020). Here we showed that the frictional properties of liquid foods influence not only subjective perceptions but also economic valuations measured in incentive-compatible auctions, naturalistic eating behavior, and neural reward responses. Our approach using biologically plausible tribology tools to quantify food textures and relate them to human perceptions, economic valuations, and neural measures could help validate the design of foods that have both healthy nutrient compositions and attractive oral-texture properties.

In conclusion, our findings show that the human OFC responded to foods in the mouth by encoding the CSF, a physical oral-texture parameter that mediated the influence of fat content on subjects' economic food valuations. Activity patterns in OFC combined sliding-friction signals with representations of subjective oiliness and economic value and explained fat preferences during naturalistic eating. Future studies could investigate how the presently identified neural mechanism for linking oral-textural properties of high-fat foods to eating behavior could contribute to overeating and obesity.
